# PPKs mediate direct signal transfer from phytochrome photoreceptors to transcription factor PIF3

**DOI:** 10.1038/ncomms15236

**Published:** 2017-05-11

**Authors:** Weimin Ni, Shou-Ling Xu, Eduardo González-Grandío, Robert J. Chalkley, Andreas F. R. Huhmer, Alma L. Burlingame, Zhi-Yong Wang, Peter H. Quail

**Affiliations:** 1 Department of Plant and Microbial Biology, University of California, Berkeley, California 94720, USA; 2 U.S. Department of Agriculture/Agriculture Research Service, Plant Gene Expression Center, Albany, California 94710, USA; 3 Department of Pharmaceutical Chemistry, University of California, San Francisco, California 94143, USA; 4 Department of Plant Biology, Carnegie Institution for Science, Stanford, California 94305, USA; 5 Thermo Fisher Scientific, San Jose, California 95134, USA

## Abstract

Upon light-induced nuclear translocation, phytochrome (phy) sensory photoreceptors interact with, and induce rapid phosphorylation and consequent ubiquitin-mediated degradation of, transcription factors, called PIFs, thereby regulating target gene expression and plant development. Nevertheless, the biochemical mechanism of phy-induced PIF phosphorylation has remained ill-defined. Here we identify a family of nuclear protein kinases, designated Photoregulatory Protein Kinases (PPK1–4; formerly called MUT9-Like Kinases (MLKs)), that interact with PIF3 and phyB in a light-induced manner *in vivo*. Genetic analyses demonstrate that the PPKs are collectively necessary for the normal light-induced phosphorylation and degradation of PIF3. PPK1 directly phosphorylates PIF3 *in vitro*, with a phosphosite pattern that strongly mimics the light-induced pattern *in vivo*. These data establish that the PPKs are directly involved in catalysing the photoactivated-phy-induced phosphorylation of PIF3 *in vivo*, and thereby are critical components of a transcriptionally centred signalling hub that pleiotropically regulates plant growth and development in response to multiple signalling pathways.

## Abstract (web summary)

Phytochrome photoreceptors mediate degradation of PIF transcription factors in the nucleus in response to light. Here Ni *et al*. identify a family of nuclear protein kinases that interact with photoactivated phytochrome B and facilitate phytochrome-induced phosphorylation and degradation of PIF3.

Plants constantly monitor ambient light conditions to adjust their growth and development for optimal photosynthesis and survival. The red and far-red wavelength information is perceived by the phytochrome (phy) family of sensory photoreceptors (phyA-E in *Arabidopsis*)^1^.

Phytochromes (phys) synthesized in seedlings germinated in darkness are mainly localized in the cytoplasm in the inactive Pr conformer. Upon red-light irradiation, the photoreceptors are converted to the active Pfr conformer, and rapidly translocate into the nucleus, where they interact directly with a subfamily of basic helix-loop-helix (bHLH) transcription factors, termed PIFs (Phytochrome-Interacting Factors; PIF1–8 (refs 2, 3)). This interaction induces phosphorylation, ubiquitination and degradation of the PIFs^2^,^4^,^5^,^6^,^7^,^8^,^9^,^10^, thereby regulating the expression of a large number of PIF target genes within minutes^11^,^12^,^13^,^14^. Although over the years there have been reports of autophosphorylation associated with biochemically isolated plant phy preparations^15^, and very recently of transphosphorylation activity towards PIF3, localized in the photosensory domain of recombinant oat phyA^16^, it has remained unclear whether these unusual activities are responsible for the multisite phosphorylation pattern that is necessary *in vivo* to trigger the normal signalling process^4^. In fact, the reported *in vitro* phy-stimulated phosphorylation of PIF3 (ref. 16) did not show the strong mobility shift on gel electrophoresis that is emblematic of normal light-induced phosphorylation *in vivo*^4^,^9^, indicating that other kinase(s) are necessary to induce multisite phosphorylation of PIF3 in the light.

Here, using mass spectrometry, we have identified a small family of protein kinases, designated photoregulatory protein kinases (PPKs) that rapidly associate with PIF3 and phyB in response to light-induced, intracellular phy activation in young *Arabidopsis* seedlings. *In vivo* and *in vitro* functional analysis of these kinases indicates that they facilitate photoactivated-phyB-induced, multisite phosphorylation and degradation of PIF3 in the nucleus.

## Results

### *In vivo* light induces a phyB-PIF3-PPK trimolecular complex

**Figure 1:**
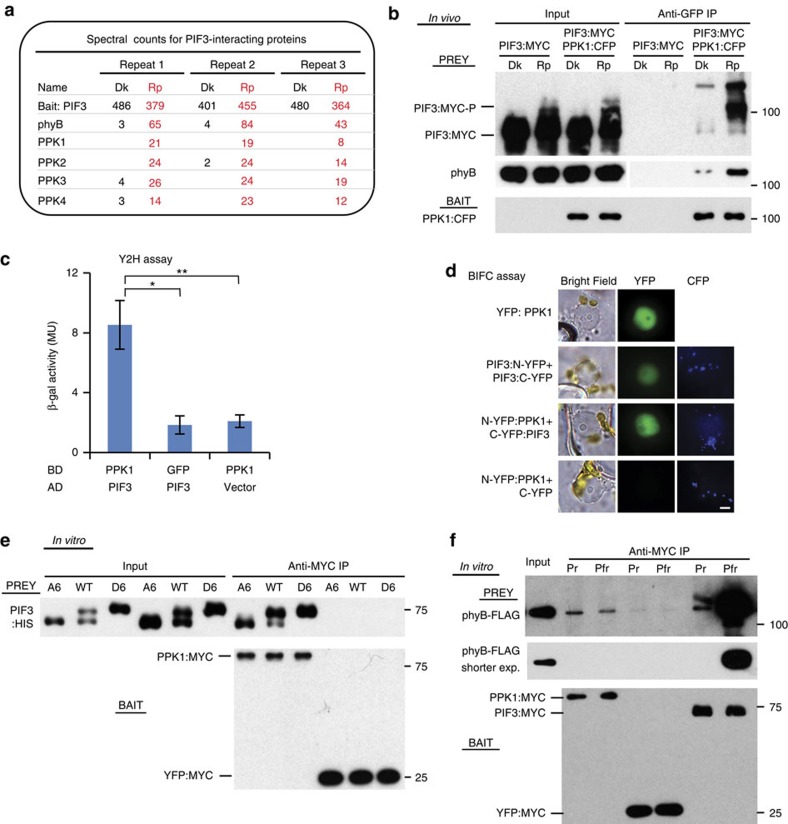
*In vivo* light promotes the interaction of PPKs with PIF3 and phyB. (**a**) PIF3-interacting proteins detected by co-immunoprecipitation (co-IP) from cell extracts and subsequent mass spectrometric analysis. Spectral counts from three biological replicates of dark (Dk)-grown and red-light-pulse (Rp)-treated seedlings, respectively. (**b**) *In vivo* light-induced interaction of PPK1 with PIF3 and phyB detected by co-IP and subsequent immunoblot analysis. Protein extracts from Dk- or Rp-treated seedlings of the indicated genotypes were immunoprecipitated with anti-GFP antibodies, to pull down CFP-tagged PPK1 as bait, and the immunoblot was probed with either anti-MYC antibody (top, Prey), anti-phyB antibody (middle, Prey) or anti-GFP antibody (bottom, Bait) to detect PIF3:MYC, phyB and PPK1:CFP, respectively. (**c**) PIF3 and PPK1 interact in yeast-2-hybrid (Y2H) assays. LexA-DNA-binding-domain-fused PPK1 or GFP were used as bait, and B42 activation-domain-fused PIF3 or empty vector were used as prey in a standard Y2H configuration. Error bars represent standard error (s.e.) from three biological replicates. *P<0.05, **P<0.01 (Student's *t*-test). (**d**) PIF3 and PPK1 interact in transient-transfection, Bimolecular Fluorescence Complementation (BiFC) assays. Light-grown *Nicotiana benthamiana* leaves were transfected with the constructs indicated and then exposed to 10 min FR light and incubated in darkness for 72 h before microscopic analysis. Constructs used: YFP:PPK1, yellow fluorescent protein (YFP) fused to PPK1 protein; N-YFP and C-YFP, N- and C-terminal domains of split mVenus210 fluorescent protein, respectively, fused (or not) to PIF3 or PPK1 proteins. All split-Venus constructs also carried the mTq2 Golgi-localized marker as a positive control for transfection. Imaging configuration: Bright field, YFP emission filter; CFP (Cyan Fluorescent Protein) emission filter. Quantification of nuclei displaying split-Venus fluorescence shown in Supplementary Fig. 2a. Scale bar, 5μm. (**e**) Interaction of *in vitro*-synthesized recombinant PPK1 and PIF3 proteins detected by co-IP assays as described in **b**, except that the PPK1 bait was tagged with MYC (PPK1:MYC), and immunoprecipitated with anti-MYC antibodies, and the PIF3 prey was tagged with HIS (PIF3:HIS). YFP:MYC bait was used as a negative control. The immunoblot was probed with either anti-HIS antibody (top, Prey) or anti-MYC antibody (bottom, Bait). WT, wild-type PIF3 sequence; A6, PIF3 variant with phospho-dead mutations; D6, PIF3 variant with phosphomimic mutations^4^,^17^. (**f**) *In vitro*-synthesized, recombinant PPK1 and phyB interact as detected by co-IP assays as described in **b**, except that the PPK1 bait was tagged with MYC (PPK1:MYC) and immunoprecipitated with anti-MYC antibodies, and the phyB prey was tagged with FLAG (phyB:FLAG). PIF3:MYC was used as a positive-control bait for light-induced phyB activation and YFP:MYC as a negative control. The immunoblot was probed with either anti-FLAG antibody (top and middle, Prey), or anti-MYC antibody (bottom, Bait). Pfr and Pr, samples were irradiated with R light only, or R followed by FR, respectively, before immunoprecipitation.

Previously we used co-immunoprecipitation (co-IP) and mass spectrometric methods to identify proteins that interact with PIF3 in a light-dependent manner *in vivo* in *Arabidopsis*, and found components of an LRB-Cullin3 E3 ligase complex, as well as all members of the phy family^17^. In addition, we also identified a small family of Casein Kinase 1-like proteins as red-light-specific, PIF3-interacting proteins in three biological replicates (Fig. 1a and Supplementary Fig. 1). We have named these proteins PPKs, PPK1, PPK2, PPK3 and PPK4, which are encoded by the At3g13670, At5g18190, At3g03940 and At2g25760 loci, respectively. These kinases were previously named MUT9-LIKE KINASEs (MLKs; MLK4, MLK1, MLK2 and MLK3, respectively) and were shown to be nuclear proteins regulating hypocotyl elongation in red light^18^,^19^. However, the signalling mechanism involved in this regulation was not determined. We propose the PPK nomenclature to reflect their central biological and biochemical functions in photosensory perception and signal transduction.

The light-dependent interaction of PPK1 with PIF3 was further confirmed here by co-IP assays using transgenic lines expressing both PIF3:MYC and PPK1:CFP. These assays involved performing co-IPs from seedling extracts to test for interactions pre-induced by light in the cell (defined here for convenience as ‘*in vivo* co-IPs'). The data show significantly higher levels of both PIF3:MYC and endogenous phyB co-precipitating from red-light-pulse (Rp)-treated than dark (Dk)-control seedling extracts when using PPK1:CFP as bait (Fig. 1b). Moreover, interestingly, there is evidence of preferential binding of PPK1 to the mobility-shifted, phosphorylated species of PIF3 in the Rp light-stimulated samples. These data indicate that photoactivated phyB induces formation of a phyB-PIF3-PPK1 trimolecular complex *in vivo* that appears to facilitate the phosphorylation of PIF3.

Interaction between PPK1 and PIF3 was also detected in yeast two-hybrid assays^20^ and in transiently transfected plant cells using a Bimolecular Fluorescence Complementation (BiFC) (or split mVenus) assay^21^ (Fig. 1c,d). The BiFC data show that PPK1 and PIF3 interact in the plant cell nucleus (Fig. 1d and Supplementary Fig. 2a). Analysis of the subnuclear dynamics of a PPK1:CFP fusion protein in response to a short red-light exposure is suggestive of partial translocation to subnuclear foci (Supplementary Fig. 2b), reminiscent of the light-induced co-localization of phy and PIF proteins in nuclear ‘speckles' or ‘photobodies'^4^,^9^,^10^. However, we have previously shown that normal phosphorylation of PIF3 is not necessary for its translocation into photobodies^4^, so it may not be unexpected that the PPKs do not obligatorily co-translocate with PIF3 and phyB into these foci. Because of the constraints imposed by the BiFC assay, requiring exposure of the cells to light during manipulations to capture the fluorescence images, we were unable to unambiguously assess whether the observed PPK1–PIF3 interaction *per se* was light-dependent or not. This question requires more refined analysis with this technology. Both *PPK1* and *PIF3* are expressed globally throughout the seedling (Supplementary Fig. 2c), in parallel to phyB^22^, providing the potential for interaction in essentially all tissues.

*In vitro* co-IP assays using PPK1:MYC synthesized in HeLa cell extracts as bait were performed to further investigate the nature of the PPK1–PIF3 interaction. We examined PPK1 binding affinity for wild-type PIF3, as well as phospho-mimic (D6) and phospho-dead (A6) PIF3 mutant variants, carrying either S-to-D or S-to-A substitutions, respectively, in the five most strongly light-induced phosphosites (six residues) previously identified *in vivo*^4^. The results show that all PIF3 variants bind PPK1 with similarly high affinity (Fig. 1e), suggesting that PPK1 directly interacts with PIF3 and that light-induced PIF3 phosphorylation is not required for this interaction. The apparent preferential binding of PPK1 to phosphorylated PIF3 species *in vivo* could reflect photoactivated-phyB stabilization of the PPK1–PIF3 interaction during active phosphorylation. Direct interaction between PPK1 and PIF3 was also demonstrated by *in vitro* co-IP assays using PPK1 as prey (Supplementary Fig. 3a), and additional assays indicate that PPK2, PPK3 and PPK4 also bind to PIF3 with affinities similar to PPK1 (Supplementary Fig. 3b). This result is consistent with potential functional redundancy among the PPKs in catalysing PIF3 phosphorylation.

Both the N-terminal kinase domain and C-terminal domain of the PPKs are highly conserved^19^. However, the full-length PPK1 and N-terminal kinase domain (PPK1N) show similar PIF3 binding affinity (Supplementary Fig. 3c), suggesting that the kinase domain is the major determinant of binding affinity with PIF3 *in vitro*.

PPK1 displays relatively weak affinity for phyB *in vitro*, with no phy-conformer preference (Fig. 1f). This result is in contrast to the well-established, robust preferential binding of PIF3 to the light-activated Pfr conformer of phyB, confirmed here, as a positive control, in parallel with the same phyB samples (Fig. 1f). Further co-IP assays confirmed the formation of a phyB-PIF3-PPK1 trimolecular complex *in vitro*, but did not detect any phyB-Pfr enhanced binding of PPK1 to PIF3 under these conditions (Supplementary Fig. 3d). These results could suggest that other molecular component(s) may be involved in the light-regulated interaction of PPK1 and PIF3 *in vivo*, or that photoactivated-phyB stabilizes the PPK1–PIF3 interaction, at least transiently, during active phosphorylation, as suggested above.

### Light-induced PIF3 phosphorylation *in vivo* requires PPKs

**Figure 2:**
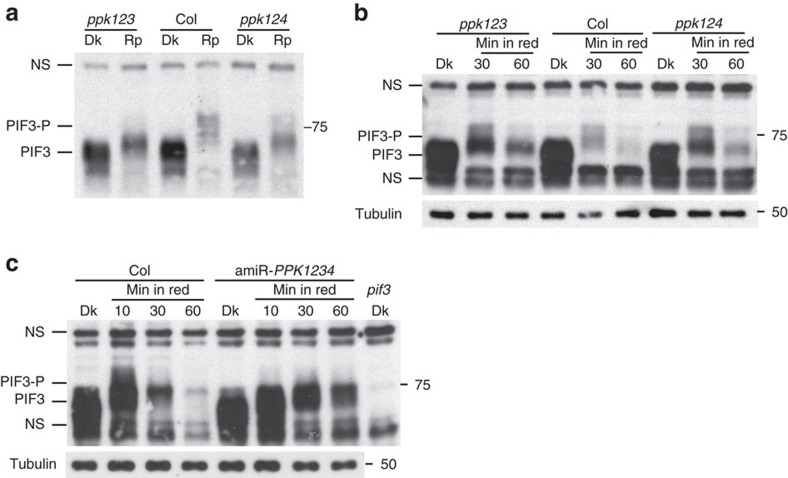
PPKs are collectively necessary for light-induced PIF3 phosphorylation and degradation *in vivo*. Both light-induced phosphorylation and degradation of endogenous PIF3 are reduced in higher order *ppk* mutants. (**a**) Rapid mobility shift (phosphorylation) and (**b**) subsequent degradation in *ppk1ppk2ppk3* (*ppk123*) and *ppk1ppk2ppk4* (*ppk124*) triple mutants compared to wild-type (Col) in response to light. (**c**) Phosphorylation and degradation in artificial microRNA *PPK1PPK2PPK3PPK4* (amiR-*PPK1234*) quadruple-knockdown mutant. Dark-grown (Dk) seedlings were irradiated with red light for 10 min (**a**, Rp), or the period indicated (**b**,**c**) before protein extraction and immunoblot analysis using anti-PIF3 antibodies. PIF3-P: phosphorylated PIF3; NS: nonspecific bands.

To examine the functional activity of the PPKs in light-induced PIF3 phosphorylation and degradation in the cell, single T-DNA insertion mutants were obtained for all four *PPK* genes (Supplementary Fig. 4). Double and triple *ppk* mutants were then generated by crossing the single mutants. However, we could not recover a quadruple *ppk* mutant, indicating that the PPKs are collectively essential for development. Both the *ppk1* single and *ppk2ppk3ppk4* (*ppk234*) triple mutants have normal PIF3 protein levels in dark-grown seedlings, and a relatively normal light-induced mobility-shift (phosphorylation) and degradation of PIF3 (Supplementary Fig. 5a), consistent with the relatively normal phenotype of these mutants for either dark- or red light-grown seedlings (Supplementary Fig. 5b). By contrast, both the *ppk1ppk2ppk3* (*ppk123*) and the *ppk1ppk2ppk4* (*ppk124*) triple mutants show a strongly lower light-induced, endogenous-PIF3 mobility shift (phosphorylation) and degradation (Fig. 2a,b), suggesting that these four PPKs are collectively necessary for the light-induced PIF3 phosphorylation and degradation. A collective function for all four PPKs is further strongly indicated by the robust reduction in PIF3 phosphorylation and degradation rate in the artificial microRNA *PPK1PPK2PPK3PPK4* (amiR-*PPK1234*) quadruple-knockdown mutant compared to Col WT in the light (Fig. 2c).

**Figure 3:**
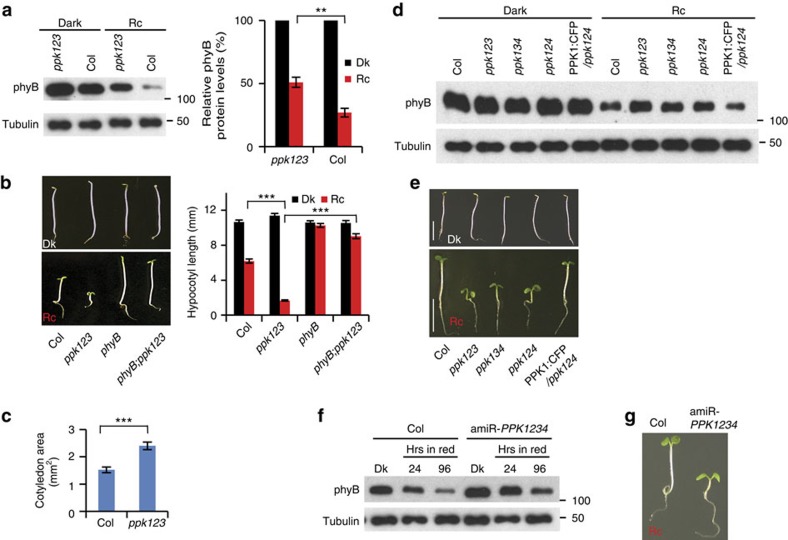
PPKs function collectively in promoting light-induced phyB degradation. (**a**) Light-induced degradation of phyB is reduced in the *ppk123* mutant. Seedlings were grown either in the dark (Dk) or continuous red right (Rc) for 4 days before protein extraction and immunoblot analysis using either anti-phyB antibody, or anti-tubulin as a loading control (left panel). Right panel shows quantification of the phyB levels relative to the tubulin protein levels from three biological replicates, expressed as a percentage of the dark-seedling levels for each genotype. Error bars represent s.e. **P<0.01 (Student's *t*-test). (**b**) A *phyB* null mutation suppresses the hypersensitive, short-hypocotyl phenotype of the *ppk123* mutant in the light (*phyB:ppk123*). Average hypocotyl lengths of 4-day Dk- or Rc-grown seedlings are shown at right. Error bars represent s.e. from three biological replicates. ***P<0.001 (Student's *t*-test). (**c**) The light-hypersensitive phenotype of the *ppk123* triple mutant includes enhanced cotyledon expansion compared to WT. Error bars represent s.e. from three biological replicates. ***P<0.001 (Student's *t*-test). (**d**) PPK1, PPK2, PPK3 and PPK4 are collectively necessary for normal PIF3 promoted phyB degradation in the light. Light-induced degradation of phyB is reduced in the *ppk123, ppk134* and *ppk124* triple-mutant backgrounds, and this reduced phyB degradation in the *ppk124* triple-mutant can be rescued by transgenic expression of PPK1:CFP (PPK1:CFP/*ppk124*). Seedlings of the indicated genotypes were grown for 4 days in the dark or continuous red light (Rc) before protein extraction and western blot analysis using an anti-phyB antibody, or anti-tubulin as a loading control. (**e**) PPKs are collectively necessary for normal hypocotyl responsiveness to light. Seedlings of the indicated genotypes were grown as in **d**. Scale bars, 5 mm. (**f**) Light-induced degradation of phyB is reduced in the amiR-*PPK1234* line. Seedling growth and western blot were done as in **d**. (**g**) The amiR-*PPK1234* line is hypersensitive to red light. Seedlings were grown for 4 days in red light.

We showed previously that light-induced multisite PIF3 phosphorylation promotes concurrent degradation of PIF3 and phyB through concurrent ubiquitination by LRB E3 ligases, thereby providing a negative-feedback attenuation mechanism that reduces phyB levels in the light^4^,^17^. Consistent with reduced light-induced PIF3 phosphorylation and degradation in the *ppk123* triple mutant, light-induced phyB degradation is also strongly reduced in the mutant (Fig. 3a). Similar to the *lrb123* triple mutant, the *ppk123* triple mutant is hypersensitive to red light, consistent with the higher levels of phyB in both these genotypes under prolonged red light (Fig. 3b,c)^17^. The two other triple-mutant combinations, *ppk124* and *ppk134*, display similar molecular and visible phenotypes to *ppk123* (Fig. 3d,e). The red-light hypersensitive phenotype of the *ppk123* triple mutant is strongly suppressed in the *phyB* mutant (Fig. 3b), consistent with the conclusion that the hypersensitive phenotype of the *ppk123* triple mutant is mainly due to reduced phyB degradation in the light. Furthermore, both the reduced light-induced phyB degradation and hypersensitive phenotype of the *ppk124* mutant can be complemented by transgenic expression of *PPK1* (Fig. 3d,e). Similarly, the reduced light-induced PIF3 phosphorylation and degradation of the amiR-*PPK1234* quadruple-knockdown mutant (Fig. 2c) is accompanied by reduced phyB degradation and a hypersensitive phenotype in red light (Fig. 3f,g).

These data strongly suggest that these four *PPK* genes are collectively necessary for normal light-induced PIF3 phosphorylation, and consequently PIF3 and phyB degradation. This proposed functional role is consistent with the parallel global spatial gene expression patterns of *PPK1, PIF3* and *PHYB* (Supplementary Fig. 2 and ref. 22) noted above. These data suggest a mechanistic basis for the recently reported involvement of the PPKs in red-light regulated hypocotyl elongation^18^. Conceptually, these results indicate that the suppressive activity of high phyB levels overrides the antagonistic promotive activity of higher PIF3 levels on hypocotyl growth, as was the case for the *lrb* mutants^17^. We suggest that this effect could result from either phyB activity independent of PIF3, from reduced intrinsic activity of PIF3, or from phyB-induced dissociation of PIF3 from its DNA binding sites. We have not attempted to define which of these, or other possible mechanisms (including the apparent trans-cellular phyB signalling recently reported^23^), might underlie this phenomenon, and so the question remains unresolved.

Interestingly, the *ppk123* mutant responds like wild type to continuous, prolonged far-red (FRc) light (Supplementary Fig. 6), indicating the absence of a function of these PPKs in phyA-regulated hypocotyl elongation under FR-high-irradiance (FR-HIR) conditions. This result is consistent with the previously reported absence of a detectable role of PIF3 in FRc-regulated hypocotyl elongation^24^,^25^. The possibility of kinases in addition to the PPKs acting in phyB-induced PIF3 phosphorylation noted above might extend to different kinases mediating phyA- and phyB-induced PIF phosphorylation^26^.

### PPK1 phosphorylates PIF3 *in vitro* mimicking *in vivo* pattern

**Figure 4:**
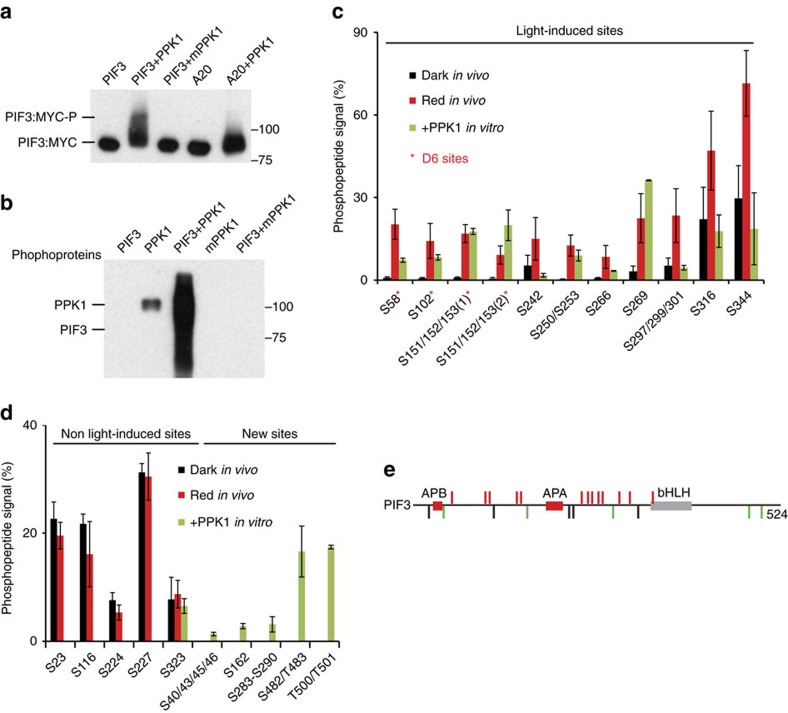
PPK1 phosphorylates PIF3 at light-inducible phosphosites *in vitro.* (**a**) PPK1 induces a strong mobility shift in PIF3 in an *in vitro* kinase assay. PPK1 and MYC-tagged PIF3 variants, affinity-purified after expression in *E. coli*, were combined under protein-kinase-assay conditions and examined for an induced, potentially phosphorylation-related, mobility-shift in PIF3 by immunoblot blot analysis using anti-MYC antibody. mPPK1: kinase-dead mutant of PPK1; A20: phospho-dead mutant of PIF3 mutated in the 20 phosphoresidues induced by light *in vivo*. (**b**) PPK1 phosphorylates PIF3 *in vitro*. Phosphoproteins from the indicated *in vitro* protein-kinase-assay combinations were detected by western blot using pIMAGO-biotin. (**c**,**d**) Mass-spectrometric analysis of *in vitro*, PPK1-catalysed phosphosites in PIF3 compared to those sites established as rapidly induced (**c**), or unaffected (**d**), by red light (Rp) *in vivo*. **d** (left), constitutively phosphorylated, non-light-induced *in vivo*, (right) not detectably phosphorylated in dark or light *in vivo*, PPK1-induced *in vitro*. Phosphopeptide Signal (%) corresponds to the percent of the residues at each site that are phosphorylated in Dk-grown or Rp-treated seedlings, or in PPK1-treated PIF3 *in vitro*. D6, strongly light-induced sites *in vivo*. Data are the means of biological repeats±s.e. (**e**) Schematic depiction of PIF3 phosphosites. Red bars represent phosphosites that are both light-induced *in vivo* and catalysed by PPK1 *in vitro*. Black bars represent sites that are constitutively phosphorylated *in vivo*, but not detectably phosphorylated by PPK1 *in vitro*, except S323. Green bars represent newly identified phosphosites catalysed by PPK1 *in vitro*. APB, active phyB-binding domain; APA, active phyA-binding domain; bHLH, basic helix-loop-helix domain.

To examine whether PPK1 can directly catalyse phosphorylation of PIF3, we performed *in vitro* kinase assays using affinity-purified recombinant PPK1 and PIF3. The data show that PPK1 induces a strong mobility shift in wild-type PIF3, but not the PIF3 A20 variant, with S/T to A mutations in the majority of the *in vivo*, light-induced phosphosites of PIF3 (ref. 4) (Fig. 4a, Supplementary Fig. 7a,b), and that this mobility-shift represents robust PPK1-catalysed transphosphorylation of the PIF3 protein, absent from the kinase-dead mutant-variant mPPK1 (Fig. 4b). High-resolution, high-accuracy mass-spectrometric analysis^27^ of this *in vitro* PPK1-phosphorylated PIF3 identified about 20 phosphorylation sites, including 13 of the 16 light-induced phosphosites that were previously identified by *in vivo* analysis (Fig. 4c). Moreover, the pattern of phosphopeptide signals for these *in vitro* sites is generally consistent with that across the light-induced sites obtained from the *in vivo* assay^4^ (Fig. 4c,e). Conversely, in addition, among the five constitutive, non-light-induced PIF3 phosphosites identified in dark-grown seedlings in our previous *in vivo* assay^4^, only one (S323) was identified in the present *in vitro* assay as phosphorylated, and the phospho-peptide signals for this, and most of the newly identified sites, are relatively low (Fig. 4d,e). Quantitative mass spectrometric analysis using a Parallel Reaction Monitoring (PRM) method further demonstrates that these 13 light-induced sites were phosphorylated by wild type, but not kinase-dead-mutant, PPK1 *in vitro* (Supplementary Fig. 7c–m). Collectively, those data indicate that the light-induced *in vivo* phosphorylation sites in PIF3 are the primary target sites of PPK1 *in vitro*.

**Figure 5:**
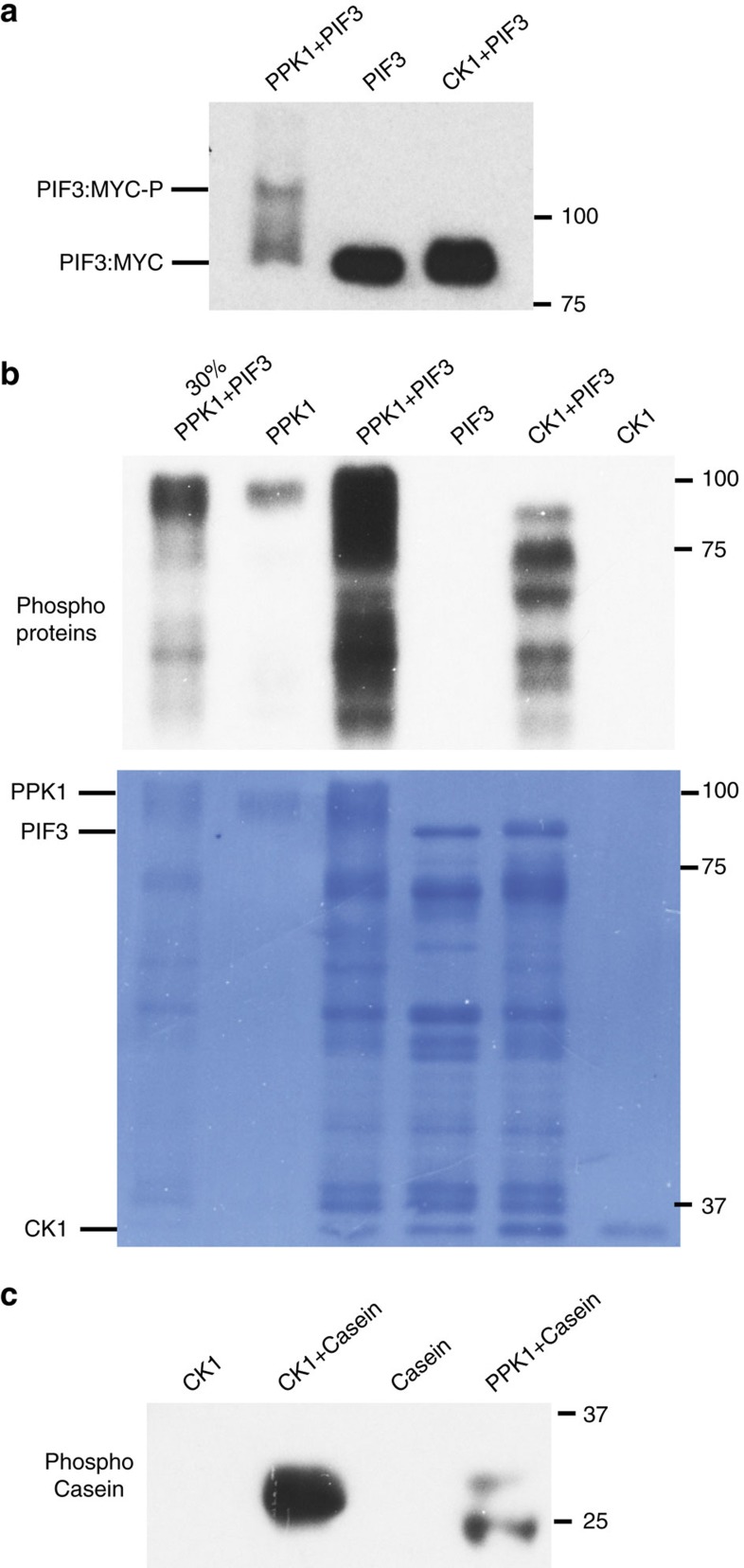
Specificity of *in vitro* phosphorylation of PIF3 by PPK1. (**a**) Unlike PPK1, CK1 does not induce a strong phosphorylation-related mobility-shift in PIF3 *in vitro*. PIF3 from the *in vitro* kinase assays as indicated was detected by immunoblot using a MYC antibody. CK1: rat Casein Kinase 1d. (**b**) PIF3 phosphorylation by CK1 is qualitatively different and quantitatively less than that by PPK1. Phosphoproteins from the indicated *in vitro* kinase assays were detected by immunoblot using pIMAGO-biotin. Signals from the kinase-only tracks indicate auto-phosphorylation activity of PPK1. A dilution (30%) of the proteins in the third lane was loaded in the first lane. Bottom panel shows the coomassie-stained membrane. Lower bands are presumptive PIF3 degradation products. (**c**) Casein phosphorylation by CK1 is greater than, and qualitatively different to, that by PPK1. Phospho-Casein from the indicated *in vitro* kinase assays was detected by immunoblot using pIMAGO-biotin.

The specificity of the *in vitro* phosphorylation of PIF3 by PPK1 was further examined by comparing it with the evolutionarily-related rat Casein Kinase 1d (CK1d). The data show that unlike PPK1, CK1d does not induce a strong mobility shift in PIF3 (Fig. 5a). Similarly, the patterns of protein-band phosphorylation catalysed by CK1d and PPK1 in the PIF3 preparations are different, indicating that PIF3 phosphorylation by CK1d is qualitatively different and quantitatively less than that by PPK1 (Fig. 5b). Conversely, although PPK1 can weakly phosphorylate casein *in vitro*, the level is much lower than for CK1d and qualitatively different (Fig. 5c).

**Figure 6:**
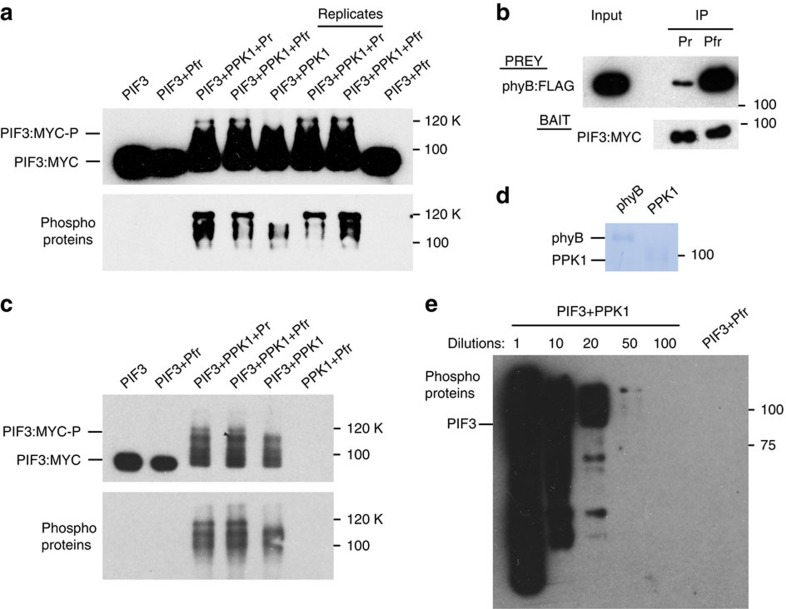
phyB stimulates PPK1-catalysed PIF3-phosphorylation in a non-light-dependent manner *in vitro* but does not detectably autonomously phosphorylate PIF3. (**a**) Mobility-shift (upper panel) and pIMAGO (lower panel) assays detect differential PPK1-catalysed phosphorylation of PIF3 *in vitro*. *In vitro* protein kinase reactions containing the protein combinations indicated were performed, followed by detection of the PIF3:MYC fusion protein by immunoblot, using anti-MYC antibodies (upper panel), and detection of phosphoproteins using pIMAGO-biotin on immunoblots (lower panel). Pr or Pfr indicate inclusion of the inactive or active conformer of phyB, respectively. Replicates indicate biological replicates of the kinase assays. (**b**) The functional activity of phyB-Pfr was verified using an established *in vitro* pull-down assay with PIF3 as bait. The immunoblot was probed with an MYC (for PIF3 bait) or FLAG (for phyB prey) antibody. (**c**) Detection of PPK1-catalysed PIF3, and potential phyB, phosphorylation *in vitro* using mobility-shift (upper panel) and pIMAGO (lower panel) assays as in **a**. (**d**) Comassie staining of phyB and PPK1 proteins used in the kinase assays in **a**. (**e**) PPK1 kinase activity is at least 50-fold greater than any potential intrinsic phyB kinase activity. Phosphoproteins from the *in vitro* kinase reactions indicated were detected by immunoblot using pIMAGO-biotin. Numbers refer to the fold-dilutions of the PIF3+PPK1 *in vitro* kinase reaction shown in **a** compared to the undiluted PIF3+Pfr reaction.

Although we did not detect any light-induced interaction of phyB-Pfr with PPK1 (Fig. 1f), or phyB-enhanced interaction of PIF3 with PPK1 (Supplementary Fig. 3d), *in vitro*, the possibility remained that light-activated phyB might enhance PPK1-catalysed phosphorylation of PIF3 in some way. However, we observed no phyB-Pfr-specific enhancement of the PPK1-induced mobility-shift of PIF3, under our *in vitro* kinase-assay conditions (Fig. 6a), despite verification that the PIF3 used in this assay bound preferentially and robustly to the phyB-Pfr conformer, as expected (Fig. 6b). Nor did we observe any mobility shift or pIMAGO-detectable phosphorylation of PIF3 in the presence of phyB-Pfr alone, without PPK1 (Fig. 6a,c,e). The limit of detection of PPK1 enzymatic activity is at a 50-fold lower protein concentration than phyB (Fig. 6d,e), indicating that PPK1 has a 50-fold higher molar capacity to phosphorylate PIF3 than any potential kinase activity intrinsic to phyB. Thus, we did not see any evidence for the recent proposal that intrinsic kinase activity in the phy molecule itself might provide ‘priming' phosphorylation of PIF3 that promotes multisite phosphorylation by other kinase(s)^16^. However, we did observe a 120-kD, conspicuously-phosphorylated, PIF3 band in the presence of both phyB and PPK1, that was absent from reactions containing PPK1 and PIF3 only (Fig. 6a,c). This band does not appear to represent PPK1-catalysed phosphorylation of phyB itself, because no such band was detected in reactions containing phyB and PPK1 only (Fig. 6c). The observation that the intensity of the phosphorylated band is equivalent in the presence of the Pr and Pfr forms of phyB indicates that the phyB molecule can partially enhance PPK1 kinase activity towards PIF3, in a conformer-independent manner. This finding could indicate, among other possibilities, undetermined differences between the molecular properties of the native and recombinant proteins used here, or the absence of some additional essential cellular factor. The possibility that light simply induces Pfr-specific nuclear translocation of otherwise constitutively active phy *in vivo* is unlikely because phy signalling in the living cell requires the Pfr conformer in the nucleus^28^.

Other previous studies have similarly proposed that constitutive (non-light-specific) phosphorylation of PIF1 and PIF4 by the protein kinases CK2 and BIN2, respectively, may prime these PIFs for subsequent light-induced, phy-mediated phosphorylation and degradation^29^,^30^. However, genetic evidence from loss of function kinase mutants is still lacking for these proposals. Here, using a kinase inhibitor and a known substrate, BZR1 (ref. 31) as a positive control, we provide evidence that the light-induced phosphorylation and degradation of PIF3 *in vivo* is not regulated by the BIN2 kinase (Supplementary Fig. 8).

## Discussion

**Figure 7:**
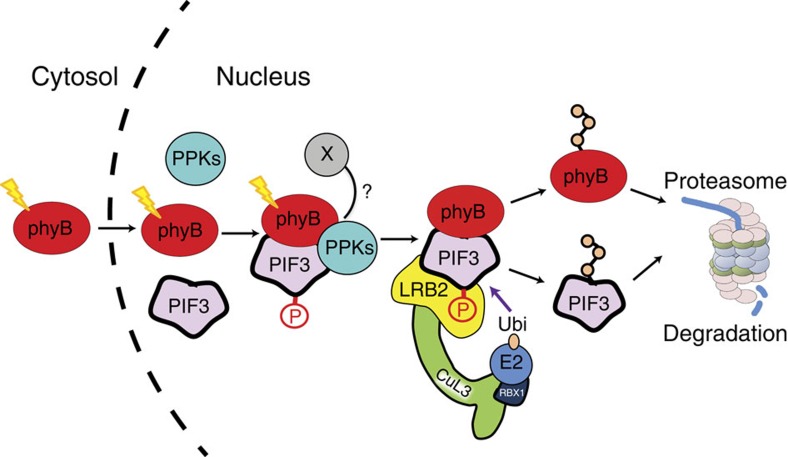
Model depicting proposed mechanism of light-induced PIF3 phosphorylation. Photoactivated phyB translocates rapidly into the nucleus and forms a trimolecular complex with PIF3 and one or more PPKs, which catalyse multisite transphosphorylation of PIF3. The LRB Cullin 3 E3 ligases then recognize phosphorylated PIF3, triggering concurrent ubiquitination and degradation of both PIF3 and phyB. X: potential additional factor contributing to Pfr-induced PIF3 phosphorylation *in vivo*, but missing from *in vitro* kinase assays performed here.

The mechanism by which photoactivated phytochrome transfers its signalling information to its signalling partner(s) in plant cells has been of central interest for decades, including the popular proposal that the phy molecule itself functions as a photoactivated S/T protein kinase, evolutionarily descended from related prokaryotic His-kinase progenitors^7^,^15^,^16^. The discovery that the Pfr-specific binding of the phy molecule to the PIFs induces rapid phosphorylation of the transcription factors in the nucleus was consistent with this possibility^4^,^5^,^6^,^8^,^9^. Investigating the alternative possibility of involvement of a third-party kinase, Bu *et al*.^30^ reported that *Arabidopsis* CK2 can phosphorylate PIF1 at multiple sites *in vitro*, and that mutation of these sites reduces the rate of light-induced PIF1 degradation *in vivo*. However, because these mutations did not alter the extent of light-induced phosphorylation *in vivo*, and loss of function *ck2* mutants enabling examination of the necessity of the CK2 family for PIF1 phosphorylation were not available, the question of the linkage of this catalytic activity to normal phy-PIF1 signalling has remained open. Our present data indicate that the PPKs are collectively necessary for light-induced, intracellular phosphorylation of PIF3, and that PPK1 has a catalytic capacity and phosphosite specificity that is very similar to the kinase(s) responsible for this *in vivo* phosphorylation. We propose a mechanistic model, therefore (Fig. 7), whereby light-activated phyB migrates into the nucleus and binds to PIF3, followed by recruitment or activation of the PPKs, which then catalyse the multisite transphosphorylation of PIF3, that is recognized by the LRB E3 ligases, leading to concurrent ubiquitination and degradation of both PIF3 and phyB^17^. The possibility that the absence of phyB-Pfr-enhanced PPK1 activity towards PIF3 in our *in vitro* kinase assays results from the absence of an additional factor(s) that is necessary for the light-stimulated phosphorylation process *in vivo*, is represented by X in Fig. 7. We see no evidence that this role is filled by kinase activity intrinsic to the phyB molecule itself towards PIF3 under our conditions, as was recently reported^16^. Based on these and previous negative findings, together with sequence evidence that the plant phys have evolved from prokaryotic progenitor His-kinases^32^, we propose that phyB functions as a pseudokinase^33^ facilitating PIF3 phosphorylation by the PPKs.

In the broader context, there is emerging evidence that the PPKs may function in multiple related signalling pathways. Notably, it has been reported that the PPKs (MLKs) are recruited to the circadian-related evening complex in green seedlings, in a photoactivated-phyB-requiring manner^18^. In addition, these kinases have been shown to regulate the activity of the blue-light photoreceptor CRY2 (ref. 34). Thus, the PPKs appear to have evolved with the potential to function in coordinating the activities of the circadian, red-light and blue-light photosensory pathways in plants through participation as components of dynamically changing multimolecular complexes.

## Methods

### Statistics

No statistical methods were used to predetermine sample size. The experiments were not randomized, and investigators were not blinded to allocation during experiments and outcome assessment.

### Plant materials and growth conditions

Three T-DNA insertion SALK lines (*PPK2* (At5g18190): SALK_026482; *PPK3* (At3g03940): SALK_000758; *PPK4* (At2g25760): SALK_017102) were obtained from ABRC. The remaining T-DNA insertion GABI-Kat line (*PPK1* (At3g13670): GABI_756G08) was obtained from NASC. The higher order mutant combinations were generated by crossing these lines.

The artificial microRNA (amiRNA) line targeting all four *PPKs* is a gift from Dr Chentao Lin.

The 35S:PIF3:MYC (ref. 4), *pPIF3*:GUS (ref. 13) and 35SBZR1:GFP (ref. 35) transgenic lines were as previously described. The 35S:PPK1:CFP transgenic lines were generated as follows: a *PPK1* cDNA was subcloned into PCR8/GW/TOPO TA cloning vector (Invitrogen) using primers specified in Supplementary Table 1, then transferred into a binary vector (pEarlygate 102) containing a 35S promoter and a CFP fusion at the C-terminus. The resulting 35S:PPK1:CFP binary vector was then transformed into the 35S:PIF3:MYC transgenic line or the *ppk124* mutant. To generate the *pPPK1*:GUS transgenic lines, the 1.9 kb *PPK1* promoter region upstream of the start codon was amplified from Col genomic DNA using primers in Supplementary Table 1, cloned into PCR8/GW/TOPO TA cloning vector, then transferred into a gateway binary vector pGWB3 containing a GUS reporter gene. Finally the binary vector was transformed into Col WT *Arabidopsis.*

Seeds were stratified for 5 days at 4 °C in darkness, and induced to germinate with 3 h white light before exposure to a terminal 5-min FRp (60 μmol m^−2^ s) on growth medium as reported^36^. For immunoprecipitation (IP) and immunoblot analysis, seedlings were grown in darkness for 2.5–3 days at 21 °C, then either kept in the dark, or given a saturating 30 s pulse of red (R) light, followed by dark incubation for a total of 10 min (Rp), or the time indicated. For seedling phenotypes, seedlings were grown either in darkness, continuous red light (6 μmol m^−2^ s) or continuous far-red (3 μmol m^−2^ s or as indicated) for 4 days at 21 °C. Measurements were done with at least 30 seedlings with three biological repeats.

### Mass spectrometric analysis of PIF3-interacting proteins

Immunopurifications of the YFP:PIF3-507 fusion protein, and mass spectrometric analyses, were performed as previously described^4^,^17^. Briefly, immunopurifications were performed to extract PIF3-interacting protein complexes from homogenates of dark-grown seedlings treated (Red) or not (Dark) with red light. The protein samples were initially separated by SDS–PAGE. The entire lane of each sample was excised and divided into about 15 segments, and subjected to reduction, alkylation and in-gel digestion with trypsin. Peptide mixtures were analysed by an LTQ–Orbitrap XL mass spectrometer (Thermo Fisher Scientific) coupled with a Nanoacquity UPLC (Waters). Three biological repeats of IP-MS were performed and spectral counts of both unique and shared peptides from each protein identified in the samples were recorded.

### Co-immunoprecipitation from seedling extracts

For co-IP using PPK1:CFP as bait, collected tissues were ground in liquid nitrogen. Proteins were then extracted into MOPS buffer (100 mM MOPS, pH7.6, 150 mM NaCl, 0.1% NP40, 1% Triton X-100, 20 mM Iodoacetamide, 1 mM phenylmethylsulfonyl fluoride, 2 μg l^−1^ aprotinin, 5 μg l^−1^ Leupeptin, 1 μg l^−1^ pepstatin, 2 × Complete protease inhibitor Cocktail and PhosStop cocktail from Roche), centrifuged and filtered through two layers of miracloth, precleared with protein A agarose beads, and then incubated with in-house rabbit anti-GFP antibodies for 1 h at 4 °C. PPK1:CFP fusion-proteins were then captured with protein A agarose beads for another hour at 4 °C, washed five times with IP buffer, transferred into a new tube before elution with boiling SDS sample buffer. The co-IP experiment was repeated three times.

### *In vitro* co-IP assays

*In vitro* co-IP assays were performed using proteins expressed in Hela cell lysates. cDNAs were amplified using the primers listed in Supplementary Table 1. Full-length or N-terminal PPK1 cDNA (encoding the kinase domain) was first cloned into pCR8/GW/TOPO vector, then transferred into an *in vitro* expression vector pT7CFE1 (Pierce) with a C-terminal HA, MYC or FLAG tag. Full-length PPK2, PPK3 and PPK4 cDNAs were cloned into the T7CFE1-CMyc vector directly using the In-Fusion HD Cloning Plus according to the manufacturer's instructions (Clontech Catalogue No: 638909). The phyB and various PIF3 vectors were as described^17^. Proteins were synthesized using the 1-step human coupled IVT kit-DNA (Pierce). Bait and prey proteins were either co-expressed, or mixed for 1 h at room temperature after expression, then diluted in PBS buffer (1 × PBS, 1 × protease inhibitor cocktail (Roche), 0.1%NP-40) and precleared with beads. The bait proteins were captured using anti-MYC antibodies (Abcam 9132) and protein G beads (Millipore). The IP products were then washed three times with PBS buffer, transferred into a new tube before elution with boiling SDS sample buffer. All *in vitro* co-IP assays were repeated at least two times.

### Immunoblots and quantification

Immunoblots and quantification of bands were performed as described^4^. Briefly, proteins were separated by SDS–PAGE, and transferred to polyvinylidene difluoride membrane. For immunoblot analysis of *in vitro* co-IP assays, horseradish peroxidase (HRP) conjugated antibody against HIS tag (GenScript The HIS-HRP, Cat. No. A00612, 0.1 μg ml^−1^), MYC tag (Cell Signaling 9B11 Cat. No. 2040S, 1:4,000 dilution), HA tag (Roche 3F10, Cat. No. 12013819001, 12 mU ml^−1^) and FLAG tag (SIGMA M2, Cat. No. A8592, 1:2,000 dilution) were used to detect the IP products. For co-IP assays from seedling extracts, mouse monoclonal antibodies against the MYC tag (Cell signaling 9B11, Cat. No. 2276, 1:5,000 dilution), GFP (Clontech, JL-8, Cat. No. 632381, 1:1,000 dilution) or phyB (B1) were used for immunodetection. For immune blots from total seedling extracts, mouse monoclonal antibody against tubulin (Sigma-Aldrich Cat. No. T9026, 1:20,000 dilution) and affinity-purified rabbit polyclonal antibody against PIF3 (ref. 9) were used for immunodetection. Anti-rabbit-HRP and anti-mouse-HRP were used as secondary antibodies (Promega), and an ECL prime chemiluminescence kit (Amersham) was used for detection. The quantification data presented in Fig. 3a were from three biologically independent replicates shown in Fig. 3a,d and Supplementary Fig. 9. Immunoblot bands were quantified using ImageJ software (NIH) and normalized to tubulin. The linearity of the signal was assessed by running a protein extract dilution curve (Supplementary Fig. 9). The relative protein level from each genotype in red light is compared with the corresponding protein level in the dark. The scanned original full-length images of gels and immunoblots are shown in Supplementary Fig. 9.

### *In vitro* kinase assays

PIF3-WT and PIF3-A20 constructs, as both N-terminal GST- and C-terminal MYC-fusions as described^4^ were used. For the kinase-dead mutant of PPK1 (mPPK1), D267 was identified as the conserved residue in the putative catalytic loop of the PPK1 kinase domain. An alignment of all the PPKs can also be found in Supplementary Fig. S1 in ref. 19. A point mutation (D267N) was introduced into *PPK1* entry clone by site-directed mutagenesis (Stratagene) using primer listed in Supplementary Table 1. Entry clones encoding the N-terminal kinase domain (PPK1-N), full-length wild-type or mutant PPK1 were then transferred into the pDEST15 gateway vector from Invitrogen for GST-fusion expression. All PIF3 and PPK1 proteins were expressed in *E. coli* (BL21 AI). For PPK1 protein expression, 2 ml overnight-grown BL21 cells expressing the desired constructs were transferred to 500 ml fresh LB medium and grown at 30 °C until OD reached 0.7–0.8. IPTG (0.1 mM) was then added into the culture to induce the protein expression overnight at 18 °C. The induction of PIF3 protein expression was as described^4^. The bacterial cells were sonicated in TBS (100 mM Tris pH 7.5, 200 mM NaCl, 2 mM DTT and protease inhibitor cocktail from Roche) with 0.5% Triton X-100 and centrifuged at 20,000*g* for 30 min to remove the insoluble cell debris. The supernatant was incubated with glutathione agarosebeads for 2 h at 4 °C. After washing with TBS, the GST-tagged proteins were eluted using 10 mM glutathione in TBS. Eluted protein was concentrated by ultrafiltration using Amicon centrifuge tubes (Millipore) with a 30 kD molecular weight cut-off. phyB:FLAG protein was expressed in Hela cell lysate and purified using FLAG antibody. Rat CK1d is from NEB. De-phosphorylated Casein is from Sigma.

*In vitro* protein kinase assays for phosphorylation of PIF3 by PPK1 were performed in a reaction mixture containing 50 mM Tris pH 7.5, 160 mM NaCl, 5 mM ATP, 10 mM MgCl_2_, 2 mM DTT, protease inhibitor cocktail from Roche, 0.5 μg PPK1 and/or 5 μg PIF3 in 10 μl. Reactions were stopped after incubation at 30 °C for 1 h unless specified. PIF3 or total phosphorylated proteins were detected by western blot analysis using anti-MYC antibody or pIMAGO (Tymora Analytical), respectively. For assays comparing PPK1 with CK1d, we used 0.5 μg PPK1 or CK1d, 2 μg PIF3 or 0.5 μg Casein in a total of 10 μl reaction volume. For *in vitro* assays with phyB, 0.25 μg of PPK1, 2 μg of PIF3 and 0.4 μg of phyB were used in a 10 μl reaction volume. The kinase assay buffer used for testing the effect of phyB was similar to the above buffer except that the concentration of NaCl was reduced to 100 mM. All *in vitro* kinase assays were repeated at least two times.

### Quantification of phosphorylation using mass spectrometry

For mass spectrometric analysis of PIF3 phosphorylation, following *in vitro* kinase reactions using wild-type and mutant PPK1, in-solution Trypsin or AspN digestion (enzyme to substrate ratio at 1:20) was carried out overnight after reduction and alkylation. Peptides were cleaned by ziptip. Data were first acquired in the Data Dependent Acquisition (DDA) mode and searched against the TAIR database (http://www.arabidopsis.org/) from December 2010, concatenated with sequence randomized version of each protein (a total of 35,386 entries) using Protein Prospector with parameters the same as described^4^. PRM^37^ acquisition using a 4 min window was scheduled with an orbitrap resolution at 60,000 (at m/z=200), AGC value 2e5 and maximum fill time of 100 ms. The isolation window for each precursor was set at 1.2 m/z unit. Both DDA and PRM data were acquired on a high resolution and accurate mass quadrupole-Orbitrap Q Exactive HF mass spectrometer^27^ coupled with Dionex UPLC (Thermo Fisher Scientific) with the same LC gradient, analytical column and normalized collision energy at 27. Quantification was carried out at both the MS1 and MS2 levels. For MS1, an extracted ion chromatogram of each peptide was generated using a 10 ppm window corresponding to the monoisotopic peak and the intensity was used for ratio calculation^4^. PRM data, which provides high-precision targeted quantification, were processed by skyline.

### Histochemical GUS staining

Histochemical GUS staining assays were performed on 2-day-old seedlings as described^13^ using a modified substrate buffer (1 × PBS (pH 7.0), 1 mM K3Fe(III)(CN)6, 0.5 mM K4Fe(II)(CN)6, 1 mM EDTA, 1% Triton X-100, 1 mg ml^−1^ Xgluc). Data from biological triplicates of two independent transgenic lines were collected, and representative images are shown for each transgene.

### Yeast-2-hybrid analysis

A full-length PPK1 cDNA in an entry vector (G11831 from ABRC) was cloned into a pEG202 gateway vector to generate the lexA BD:PPK1 construct. A full-length PIF3 entry vector^9^ was cloned into a JG4.5 gateway vector to generate the B42AD:PIF3 construct. The BD:PIF3 and BD:GFP constructs were described in reference^4^. All constructs were transformed into yeast stain EGY48. Yeast-two hybrid interaction assays were performed using a standard liquid ONPG assay as described in Clontech Yeast Protocols Handbook.

### BiFC transient transfection assays in *Nicotiana* leaves

Full-length PPK1 cDNA in a gateway entry vector (G11831 from ABRC) was cloned into the pEarleyGate 104 vector to generate the 35S:YFP:PPK1 vector. For BiFC assays, the vector system described in ref. 38 was used. This system employs an optimized single vector BiFC construct, which utilizes monomeric Venus (mVenus) split at residue 210, and contains an integrated mTurquoise2 (mTq2) marker to precisely identify transformed cells in order to distinguish true split-Venus negatives. Full-length PIF3 and PPK1 cDNAs were amplified using primers specified in Supplementary Table 1 and cloned into pDOE5 for PIF3–PIF3 interactions, and into pDOE8 for PIF3–PPK1 interactions. Plasmids were transformed into *Agrobacterium tumefaciens* GV3101. *Nicotiana benthamiana* plants were grown in long day conditions (16 h white light (120 μmol m^–2^ s)/8 h dark) at 22 °C. Agroinfiltration was performed as described in ref. 39, using a final OD_600_ of 0.1 for each culture. 35S:p19 (ref. 40) was co-infiltrated with each construct. After inoculation, plants were given a 10 min FRp (235 μmol m^−2^ s) and kept in the dark. Infiltrated *Nicotiana* leaves were tested 3 days post-inoculation. The abaxial epidermis of test leaves was peeled, and images were taken using a Leica DM4000 B microscope fitted with a YFP filter. At least five leaves from different plants were examined for each construct. For signal quantification, samples were examined with a × 40 magnitude objective and nuclei with a fluorescent signal were counted in five microscopic fields per construct. The number of nuclei displaying fluorescence in each field was expressed as a percentage of the total number of nuclei visible in that field (Supplementary Fig. 2a).

### Epifluorescence analysis of PPK1:CFP

Epifluorescence microscopy analysis of PPK1:CFP in the hypocotyl cell of a PPK1:CFP (*ppk124*) transgenic line were performed by using an Axiovert 200 microscope (Zeiss, Oberkochen, Germany) with excitation and detection of the CFP fluorophore. Images were recorded with a digital Axiocam camera (Zeiss) and processed for optimal presentation using the Adobe Photoshop software package.

### Data availability

The authors declare that all data supporting the findings of this study are available within the manuscript and its supplementary files or are available from the corresponding author on request.

## Additional information

**How to cite this article:** Ni, W. *et al*. PPKs mediate direct signal transfer from phytochrome photoreceptors to transcription factor PIF3. *Nat. Commun.* **8**, 15236 doi: 10.1038/ncomms15236 (2017).

**Publisher's note:** Springer Nature remains neutral with regard to jurisdictional claims in published maps and institutional affiliations.

## Supplementary Material

Supplementary Information Supplementary Figures and Supplementary Table
