# Supplementary material for: PPKs mediate direct signal transfer from phytochrome photoreceptors to transcription factor PIF3
Source: Nat Commun. 2017 May 11;8:15236. doi: 10.1038/ncomms15236 (PMC5437280; doi:10.1038/ncomms15236)
Supplement: Supplementary Information — Supplementary Figures and Supplementary Table [file ncomms15236-s1.pdf]

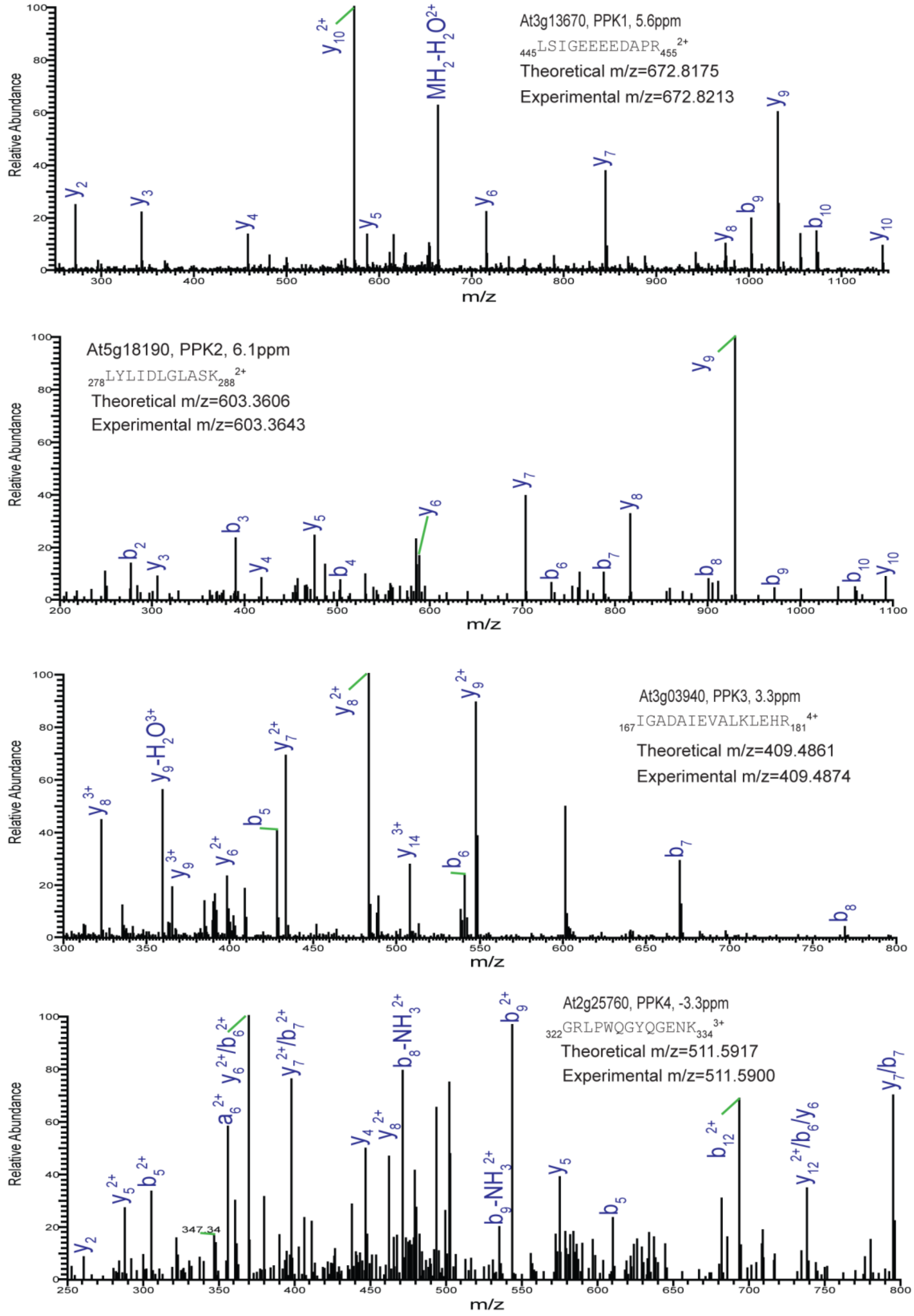

Supplementary Figure 1 | Collision-induced dissociation (CID) mass spectra of peptides from PPK1, PPK2, PPK3 and PPK4 respectively.

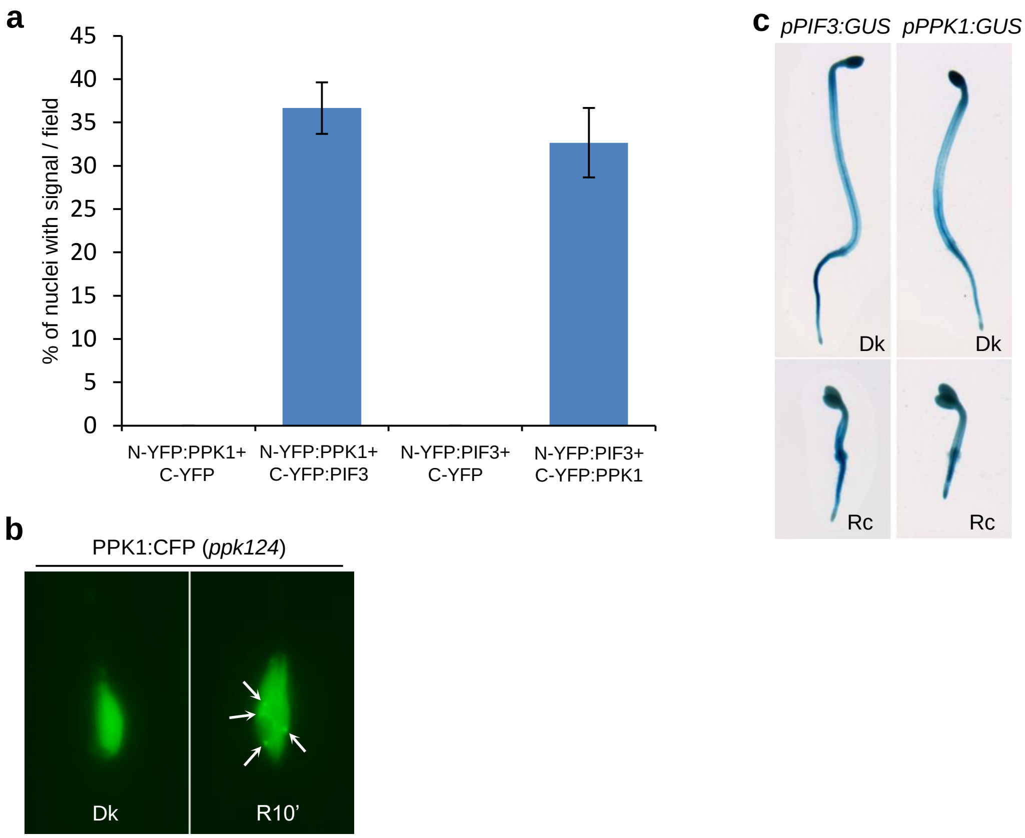

Supplementary Figure 2 | Intranuclear interaction and overlapping, seedling-wide expression of PPK1 and PIF3. a, PIF3 and PPK1 interact in transient-transfection, Bimolecular Fluorescence Complementation (BiFC) assays. Quantification of BiFC assays in *Nicotiana* leaves as in Figure 1d. Nuclei displaying a split-mVenus YFP fluorescence signal were counted in five microscope fields, at 40X for each construct, and expressed as a percentage of the total number of nuclei visible in that field. Constructs used: N-YFP and C-YFP, N- and C-terminal domains of split mVenus210 fluorescent protein, respectively, fused (or not) to PIF3 or PPK1 proteins, and infiltrated in two reciprocal configurations. Data are shown as mean ± SEM. **b,** PPK1 subnuclear localization analysis in the PPK1:CFP (*ppk124*) transgenic line as in Figure 3. Representative epifluorescent images of CFP fluorescence in hypocotyl-cell nuclei are shown. Seedlings were grown for 3 days in the dark, and then either maintained in the dark (Dk), or given a saturating red-light pulse and returned to the darkness for 10 min (R10’) before imaging. Arrows indicate apparent light-induced nuclear speckles. **c,** *PPK1* displays a gene expression pattern similar to *PIF3* both in the dark and light. Representative images of histochemical staining of GUS activity in 2-day-old transgenic seedlings grown in the dark (Dk) or continuous red light (Rc). The GUS reporter gene was driven by the *PIF3* (left panels) and *PPK1* (right panels) promoters, respectively.

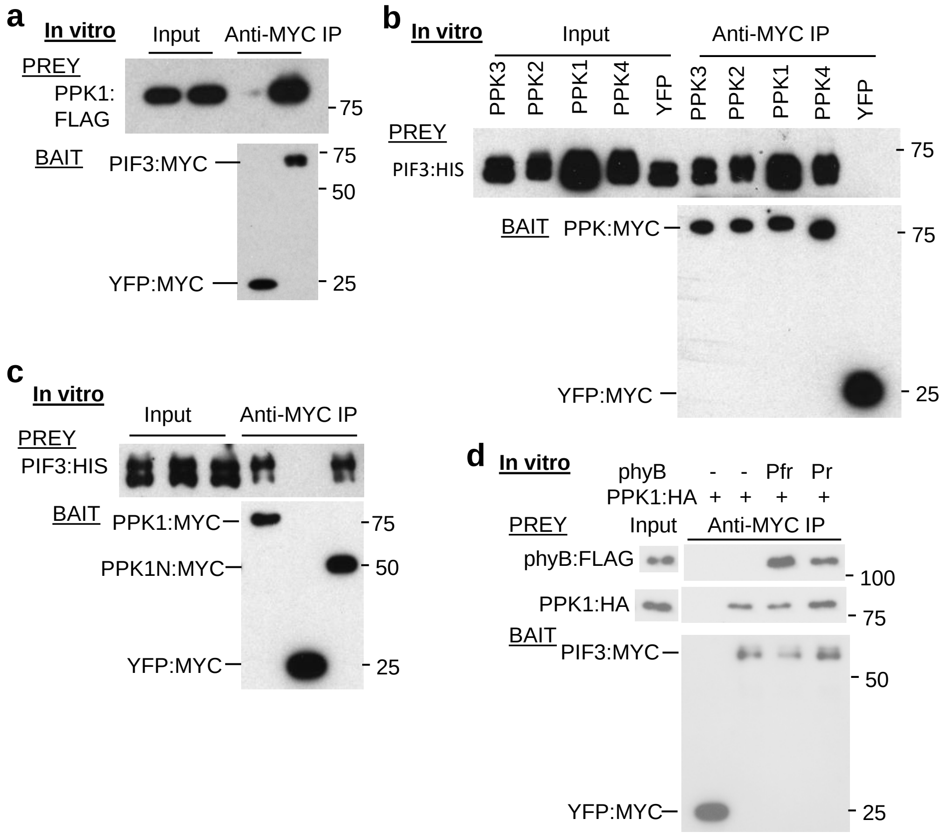

Supplementary Figure 3 | PIF3 interacts with PPK1, PPK2, PPK3 and PPK4 in vitro.
a, PIF3 interacts with PPK1 in vitro. PPK1:FLAG was co-expressed with PIF3:MYC or YFP:MYC (control) in a Hela cell lysate, then immunoprecipitated with anti-MYC antibodies. Bait and prey proteins were detected by immunoblot using anti-MYC (bottom) and anti-FLAG antibody (top), respectively. **b,** PIF3 interacts with PPKs in vitro. PIF3:HIS was co-expressed with PPK3-, PPK2-, PPK1 or PPK4-MYC-fusion proteins, then immunoprecipitated with anti-MYC antibodies. Proteins were analyzed by immunoblot using anti-HIS antibody (top panel, Prey) or anti-MYC antibody (bottom panel, Bait). YFP:MYC was used as a negative control. **c,** PIF3 interacts with the N-terminal kinase domain of PPK1 (PPK1N) in vitro as detected by co-IP assay as described in **b**. **d**, phyB, PIF3 and PPK1 form a trimolecular complex in vitro but phyB-Pfr binding does not enhance PPK1 and PIF3 interaction in vitro. In vitro expressed PPK1 and PIF3 were incubated in the absence (-) or presence of phyB (Pfr or Pr conformer), then immunoprecipitated with anti-MYC antibodies. Proteins were analyzed by immunoblot using anti-FLAG antibody (top panel, phyB Prey), anti-HA antibody (middle panel, PPK1 Prey), or anti-MYC antibody (bottom panel, PIF3 Bait). YFP:MYC was used as a negative control.

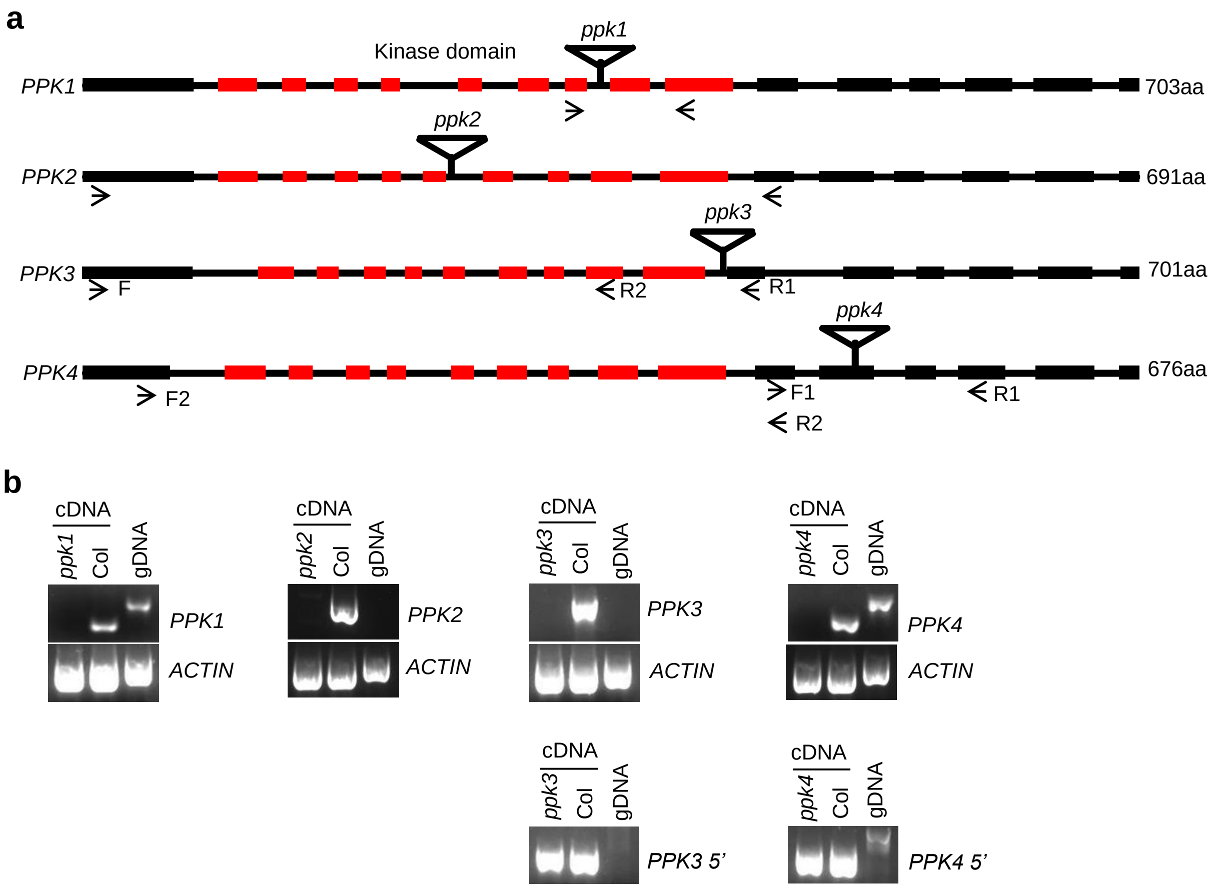

Supplementary Figure 4 | Characterization of *ppk* mutants.
a, Cartoon of *ppk* T-DNA-insertion mutants. Boxes indicate the exons in the four *PPK* genes. Red area represents the kinase domain, triangles indicate the positions of T-DNA insertions, and arrow heads refer to the positions of primers used for RT-PCR analysis in panel **(b)**. **b,** RT-PCR analysis of *ppk* mutants. Top, primers specific for the full-length open reading frame corresponding to the gene indicated were used to amplify the respective gene from cDNA derived from the indicated line or from wild-type genomic DNA (gDNA). The *ACTIN* gene was amplified as a control. Bottom, primers specific for a portion of the *PPK3* or *PPK4* gene upstreamto the site of the T-DNA insertion, were used to amplify cDNA by RT-PCR for the indicated line. Primer sequences can be found in Supplementary Table 1.

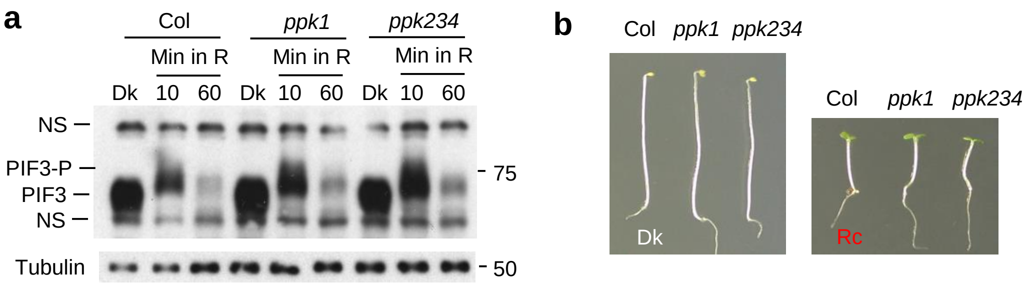

Supplementary Figure 5 | PPK1, PPK2, PPK3 and PPK4 function collectively in promoting light-induced PIF3 phosphorylation and degradation.

a, Light-induced PIF3 phosphorylation and degradation is normal in the *ppk1* single and *ppk2ppk3ppk4* triple-mutants (*ppk234*). Dark-grown (Dk) seedlings of the indicated genotypes were irradiated with red light for the period indicated before protein extraction and Western blot analysis using anti-PIF3 antibodies. NS: nonspecific bands. PIF3-P, phosphorylated PIF3. **b,** the *ppk1* single and *ppk234* triple-mutant seedlings have normal phenotypes. Seedlings of the indicated genotypes were grown for 4 days in the dark (Dk) or continuous red light (Rc).

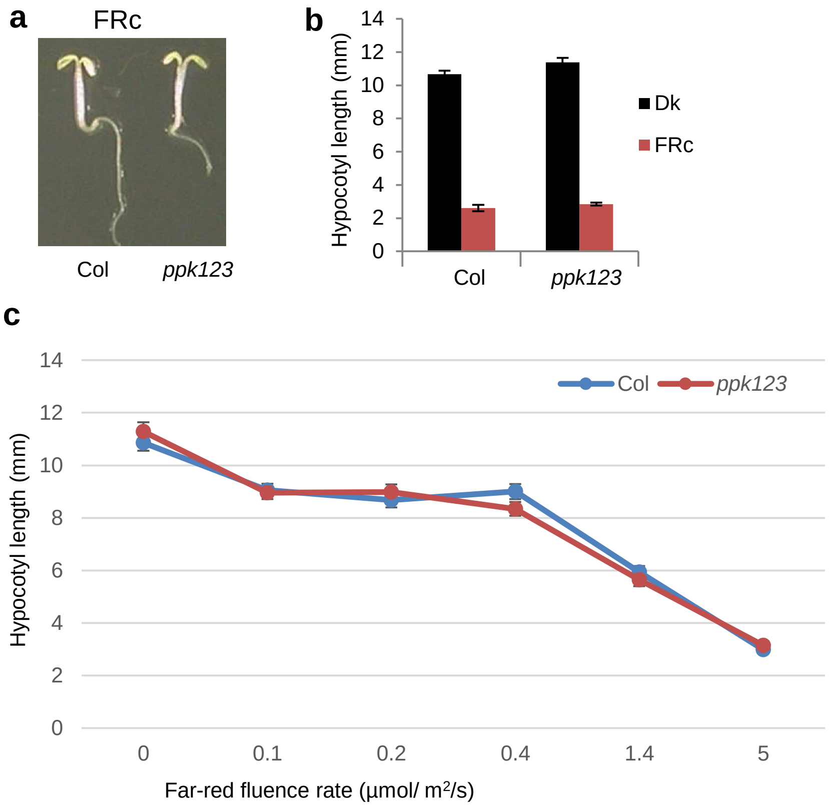

Supplementary Figure 6 | The *ppk123* triple-mutant responds normally to prolonged, continuous far-red light (FRc). a, The *ppk123* triple-mutant seedlings have phenotypes indistinguishable from WT in FRc, at 3 micromole/m^2/s^1. **b,** The *ppk123* triple-mutant seedlings respond normally to both dark and FRc. Seedlings of the indicated genotypes were grown for 4 days in the dark (Dk) or continuous far-red light (FRc) , at 3 micromole/m^2/s^1. Error bars represent SE. **c,** Far-red fluence-rate response curve for hypocotyl elongation in *ppk123* triple-mutant seedlings is normal. Seedlings of the indicated genotypes were grown for 4 days in the dark (Dk) or continuous far-red light (FRc). Error bars represent SE.

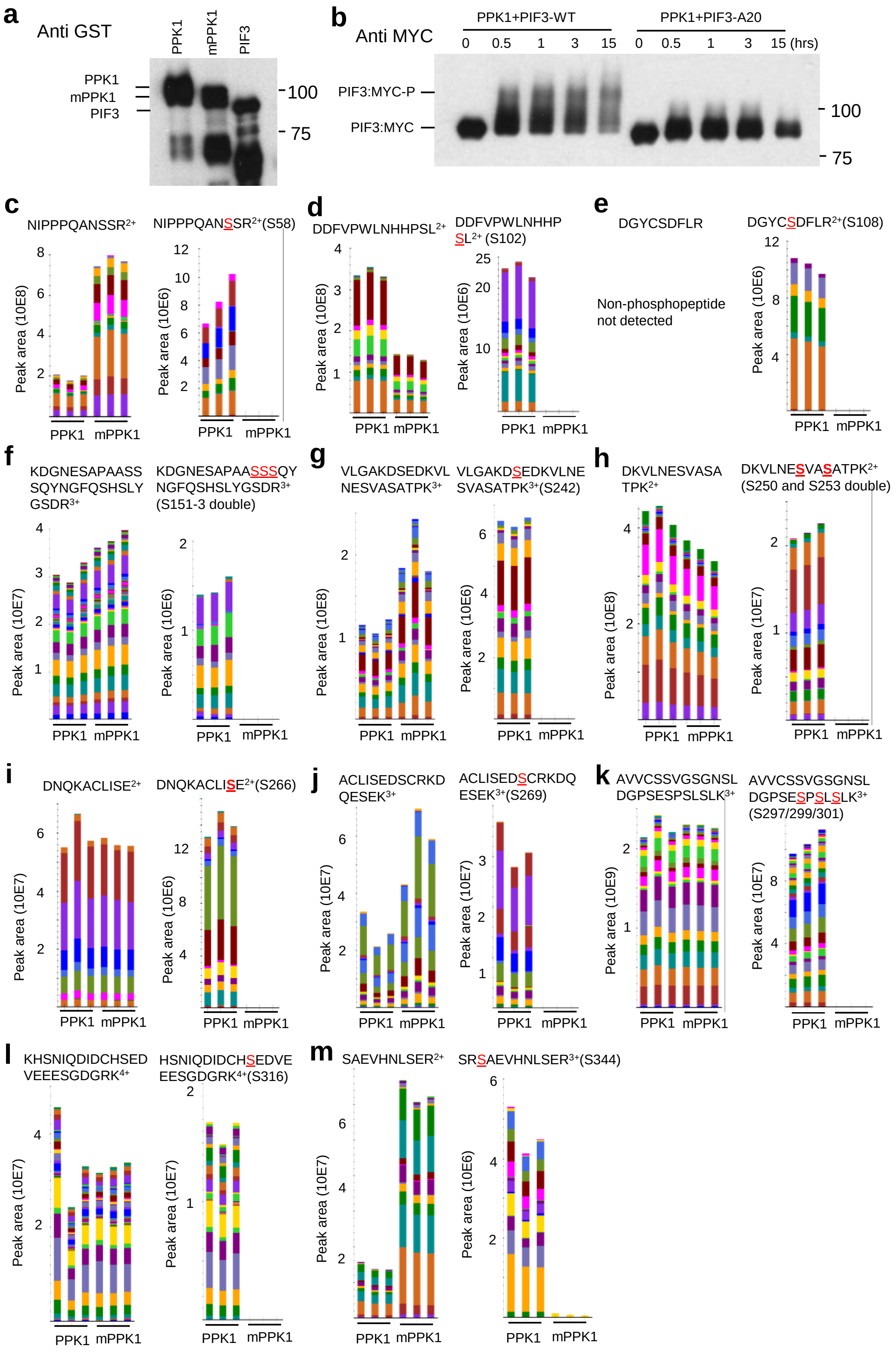

Supplementary Figure 7

Supplementary Figure 7 | PPK1 alone phosphorylates PIF3 in vitro at previously-identified light-inducible phosphosites.

a, Western blot analysis, using an anti-GST antibody, of N-terminal GST-fusion proteins purified from E.coli for in vitro kinase assays. Top band in each lane represents full-length proteins, lower bands are partially degraded products. **b,** PPK1 induces a strong phosphorylation-related, mobility-shift in wild type, but not phosphosite mutant-variant of PIF3 in vitro. In vitro kinase assays were performed over the time-course indicated using purified GST-fusion proteins from **(a)**. Full-length PIF3 proteins were detected by immunoblot using an antibody against the MYC epitope fused at the C-terminus. A20: phosphosite-dead mutant of PIF3 mutated in the 20 phosphoresidues induced by light in vivo. **c-m,** Mass spectrometric quantification of PIF3 phosphorylation from the in vitro kinase assays with PPK1 or mPPK1 (kinase-dead mutant of PPK1) as in Fig. 4a**,** using the Parallel Reaction Monitoring (PRM) method. The total integrated fragment ion signal for each peptide containing the light-induced phosphorylation site is plotted as a bar graph. The contribution from each individual fragment ion is displayed as a different color in the bars. Data from three technical repeats of the non-phosphopeptide (left) and phosphopeptide (right) fragments are shown in each panel.

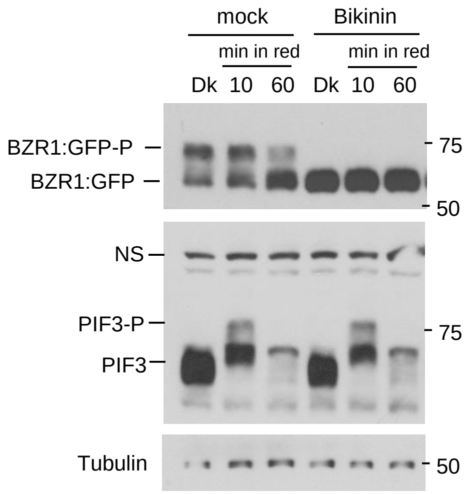

Supplementary Figure 8 | BIN2 kinase is not required for the light-induced PIF3 phosphorylation and degradation in vivo.
Three-day-old dark-grown transgenic seedlings expressing a BZR1:GFP transgene were pretreated with the BIN2-kinase inhibitor, Bikinin, or mock for 1 hour before red light treatment for the period indicated. Extracted Proteins were analyzed by immunoblot using anti-GFP antibody (for BZR1, top panel), or anti-PIF3 antibodies (middle panel), or anti-tubulin as a loading control (bottom panel).

Fig. 1b

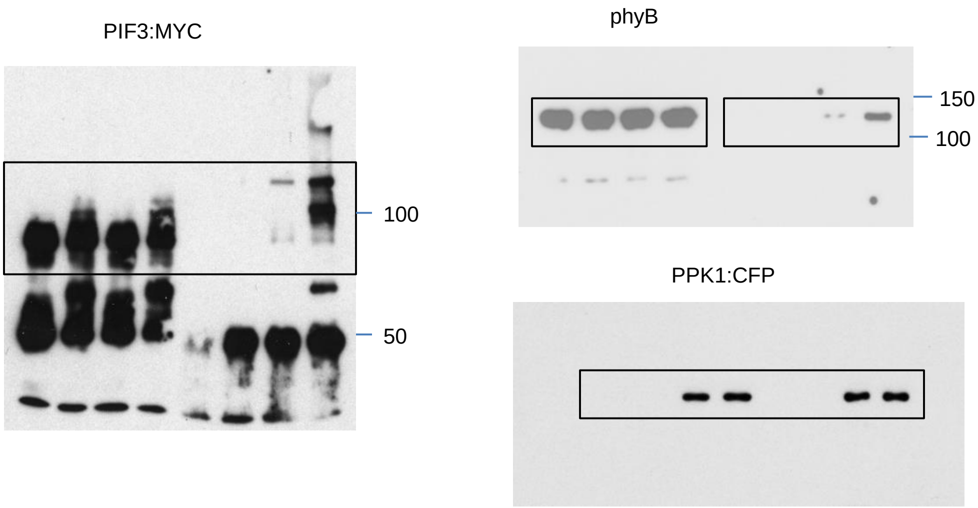

Fig. 1e

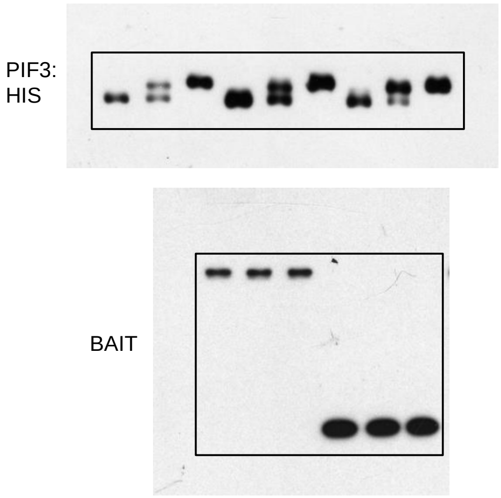

Fig. 1f

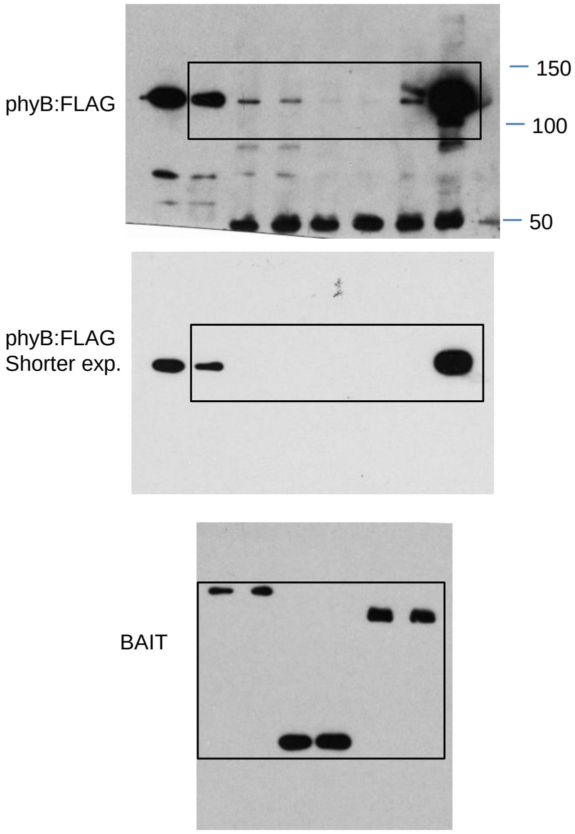

Supplementary Figure 9 | Scanned original full-length images of gels and immunoblots.

Fig. 2a

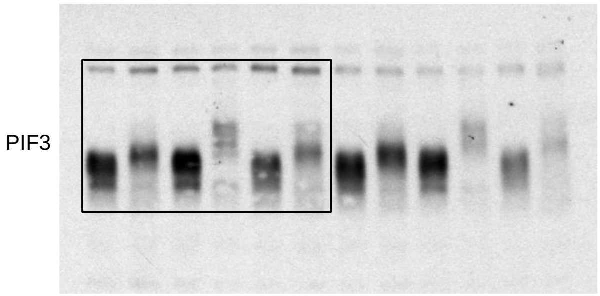

Fig. 2b

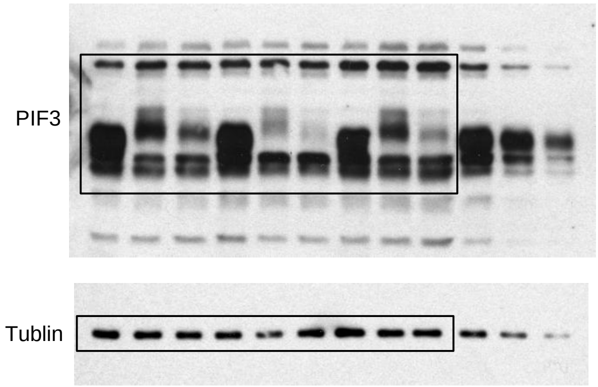

Fig. 2c

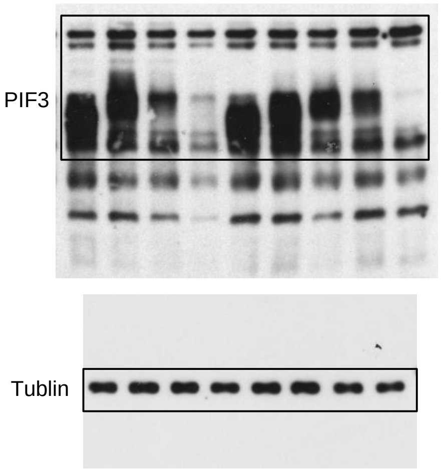

Supplementary Figure 9 . (continued)

Fig. 3a

phyB Tublin

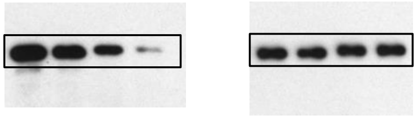

Fig. 3d

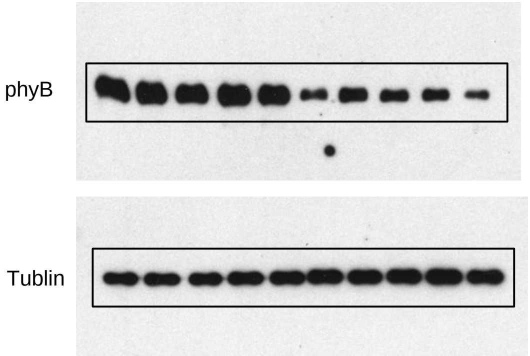

Fig. 3f

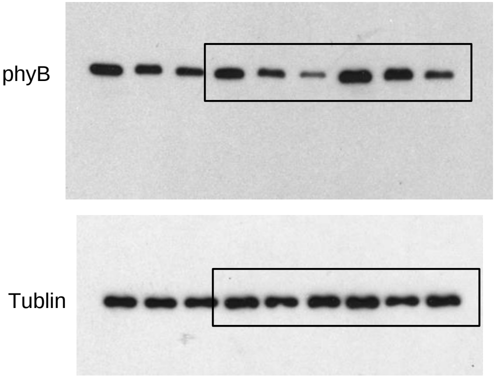

Third replicate for quantification in Fig. 3a, right-hand panel

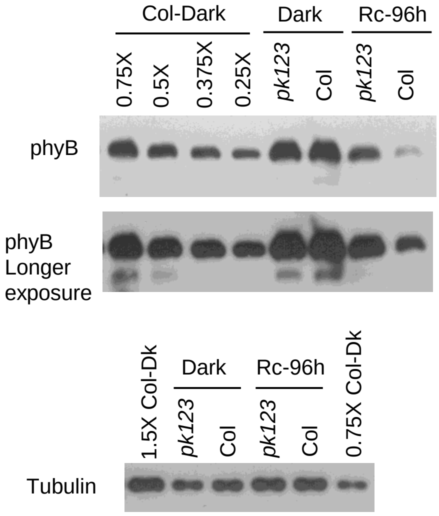

Supplementary Figure 9 . (continued)

Fig. 4a

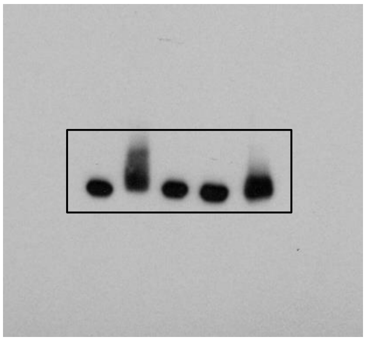

Fig. 4b

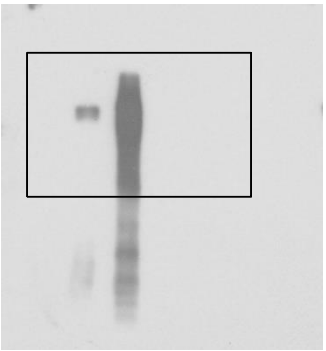

Supplementary Figure 9 . (continued)

Fig 5a

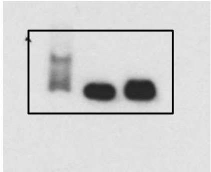

Fig 5b

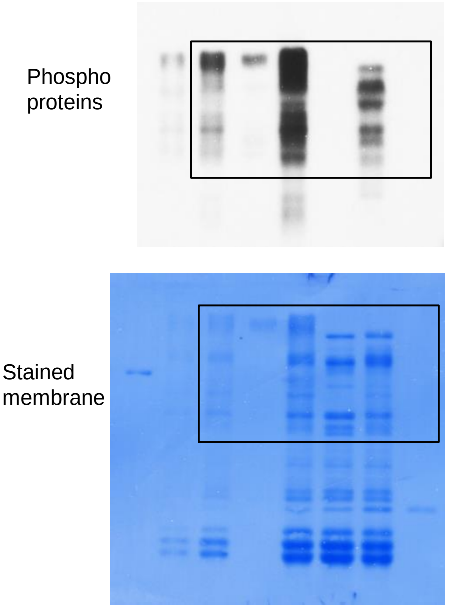

Fig 5c

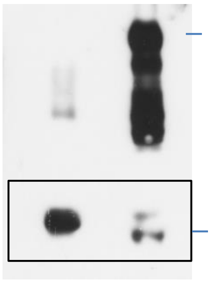

Supplementary Figure 9 . (continued)

Fig. 6a

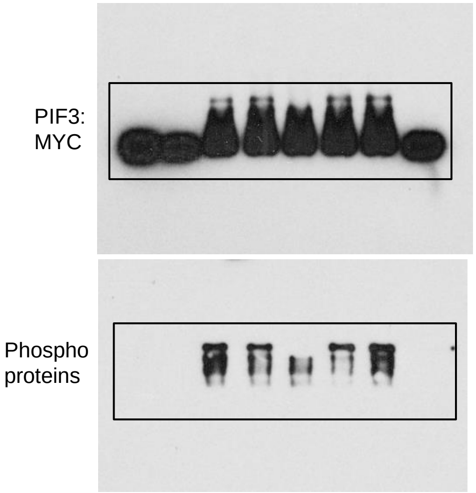

Fig. 6b

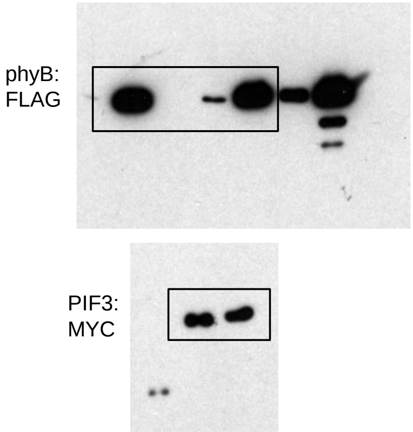

Fig. 6c

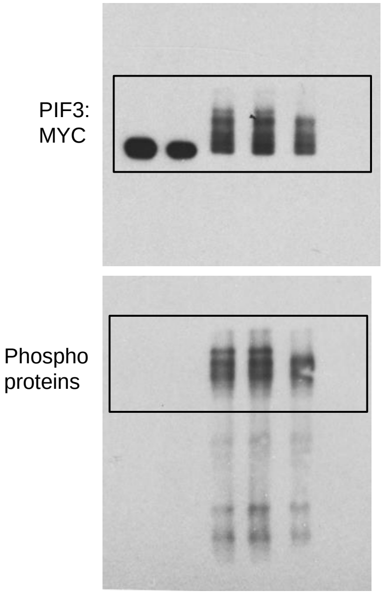

Fig. 6d

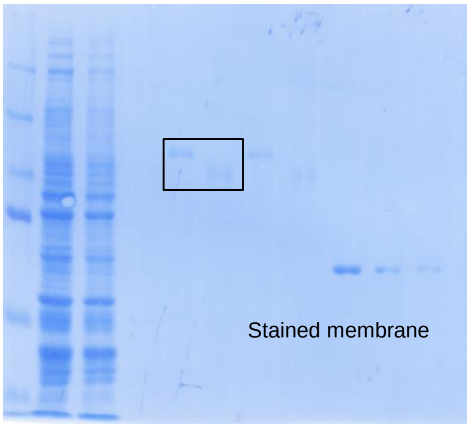

Fig. 6e

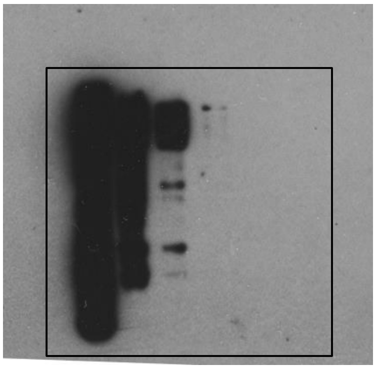

Supplementary Figure 9 . (continued)

Supplementary Table 1 | Primer sequences

Primer name	Primer sequence
Genotyping	
PPK3gF	TCCTCGAGGCAGTTACGAAT
PPK3gR	GCCAAAATAAATGCAAAGCC
PPK3-F4	TCATGCATTGTTCATTCCCT
LBa1	TGGTTCACGTAGTGGGCCATCG
PPK2-F3	GCAAAGGATGCAATTTTGGT
PPK2-R2	GCCAAGAAAATGCAAATGGT
PPK2-F1	atgccagagttaagaagtggagc
PPK1-F4	TTCAGTTATGTGCATGGGGA
PPK1-R4	ATGTCACCTTTTGAGCACCC
PPK1 T-DNA	ATATTGACCATCATACTCATTGC
PPK4-F2	TCCTGGCAACTTTCCTTGTC
PPK4-R2:	AACCACGAATGAGTTCCCAC
RT-PCR	
PPK3RT-F	GATCGAGACGCCTTGACGAGC
PPK3RT-R1	TAGACTGAAATCCACTGTGTG
PPK3RT-R2	ACGACCAAGATGTGCATGGCA
PPK2RT-F	AACCTAGCCCTCAGGTGAC
PPK2RT-R	AGACTGAGATCCACTGACAA
PPK1RT-F	AGTGGAGAGAAGGTGGTAGCGGAC
PPK1RT-R	GCGATGTTGCCATCTTCTTTTTGC
PPK4RT-F1	TGGATCAGCATTTACAGTGCTCACAGAC
PPK4RT-R1	TCCACTTGAAGGGAAAAGAATCACTGAC
PPK4RT-F2	CTGATGATGAAGGAAACGCTCC
PPK4RT-R2	GTCTGTGAGCACTGTAAATGCTG
Actin2RT-F	GTTGGGATGAACCAGAAGGA
Actin2RT-R	GAACCACCGATCCAGACACT
Promoter GUS	
PPK1-Pr-F4	ATGTCGAGGCTCGCACCAAG
PPK1-Pr-R:	AGCTCAAACCCCATGCAATCG
Pull down	
NdeI-PPK1F	catatgATGCCGGAGC TTCGCCGTGG
SalI-PPK1R	gtcgacAGATACAGTTCGGCCATAGC
SalI-HA-PPK1-R	gtcgacGGCGTAGTCGGGCACGTCGTAGGGGTAAGATACAGTTCGGCCATAGC
SalPPK1N-R	gtcgacTTCGCCCAAGAGGTCTTGA
NdeI-PPK3F	TGGCCACCACCCATATGCCAGAGT TAAGAAGTGG
SalI-PPK3R	GTGCGGCCGCGTCGACGCAAACTGTCCGACCATAGC
SalI-PPK2R	GTGCGGCCGCGTCGAC GCAAACTGTCCTCCCAAAGC
NdeI-PPK4F	TGGCCACCACCCATATGCCTGAGCTGCGTAGCAA
SalI-PPK4R	GTGCGGCCGCGTCGAC TGACACAGTTCGACCATAAC
In vitro kinase assay	
PPK1-D267N	ggttatgtgcatgggaatgttaagccagaa
BiFC	
AscI-PIF3	TTggcgcgccATGCCTCTGTTTGAGCTTTTC
PIF3-AvrII	AGAcctaggccCGACGATCCACAAAACTGATC
AscI-PPK1	TTggcgcgccATGCCGGAGCTTCGCCGTGG
PPK1-SpeI	GGactagtAGATACAGTTCGGCCATAGC
SanDI-PIF3	GCATGgggtccCCTCTGTTTGAGCTTTTCAG
PIF3-BspEI	TGTGtccggaCGACGATCCACAAAACTGATCA
SanDI-PPK1	GCATGgggtccCCGGAGCTTCGCCGTGGAG
PPK1-BspEI	TGTGtccggaAGATACAGTTCGGCCATAGC
